# Targeting hormone refractory prostate cancer by *in vivo* selected DNA libraries in an orthotopic xenograft mouse model

**DOI:** 10.1038/s41598-019-41460-2

**Published:** 2019-03-21

**Authors:** Laia Civit, Ioanna Theodorou, Franziska Frey, Holger Weber, Andreas Lingnau, Carsten Gröber, Michael Blank, Chloé Dambrune, James Stunden, Marc Beyer, Joachim Schultze, Eicke Latz, Frédéric Ducongé, Michael H. G. Kubbutat, Günter Mayer

**Affiliations:** 1 Chemical Biology and Chemical Genetics, Life and Medical Sciences (LIMES) Institute, University of Bonn, Gerhard-Domagk-Str. 1, 53121 Bonn, Germany; 2 CEA, DRT, Institut de biologie François-Jacob, Molecular Imaging Research Center (MIRCen), UMR CNRS 9199, 18 Route du Panorama, 92260 Roses, France; 3 KTB Tumorforschungsgesellschaft mbH, Research Division ProQinase, Breisacher Str. 117, 79106 Freiburg, Germany; 4 AptaIT GmbH, Am Klopferspitz 19a, 82152 Planegg, Martinsried Germany; 5 Institute of Innate Immunity, University Hospital Bonn, Sigmund-Freud-Str. 25, 53127 Bonn, Germany; 6 Genomics and Immunoregulation, Life and Medical Sciences (LIMES) Institute, University of Bonn, Carl-Troll-Straße 31, 53115 Bonn, Germany; 7 Platform for Single Cell Genomics and Epigenomics at the DZNE and the University of Bonn, Sigmund-Freud-Str. 27, 53127 Bonn, Germany; 8 Molecular Immunology in Neurodegeneration, German Center for Neurodegenerative Diseases (DZNE), Sigmund-Freud-Str. 27, 53127 Bonn, Germany; 9 Center of Aptamer Research and Development (CARD), University of Bonn, Gerhard-Domagk Str. 1, 53121 Bonn, Germany; 10 Present Address: ProQinase GmbH, Breisacher Straße 117, 79106 Freiburg, Germany; 11 Present Address: Genmab B.V., Yalelaan 60, 3584 CM Utrecht, The Netherlands

## Abstract

The targeting of specific tissue is a major challenge for the effective use of therapeutics and agents mediating this targeting are strongly demanded. We report here on an *in vivo* selection technology that enables the *de novo* identification of pegylated DNA aptamers pursuing tissue sites harbouring a hormone refractory prostate tumour. To this end, two libraries, one of which bearing an 11 kDa polyethylene glycol (PEG) modification, were used in an orthotopic xenograft prostate tumour mouse model for the selection process. Next-generation sequencing revealed an *in vivo* enriched pegylated but not a naïve DNA aptamer recognising prostate cancer tissue implanted either subcutaneous or orthotopically in mice. This aptamer represents a valuable and cost-effective tool for the development of targeted therapies for prostate cancer. The described selection strategy and its analysis is not limited to prostate cancer but will be adaptable to various tissues, tumours, and metastases. This opens the path towards DNA aptamers being experimentally and clinically engaged as molecules for developing targeted therapy strategies.

## Introduction

Prostate cancer is the most common cancer among men, with approximately 1.1 million new cases diagnosed per year worldwide^1^. It accounts for the second most incidence of cancer-related death although the 5-year survival rate is over 80%. Especially, hormone refractory prostate cancer is not curable and targeted therapies are yet not available. Hormone refractory prostate tumour cells likely metastasise to distal sites accounting for poor prognosis and survival of patients^2^. For prostate tumours not responding to initial hormone therapy, chemotherapy with docetaxel (taxotere) results in an improved survival of patients^3^. Alternative therapies are immunotherapy with sipuleucel-T, an autologous dendritic cell- based cancer vaccine, agents interfering with androgen signalling, i.e. abiraterone or enzalutamide, and radiopharmaceutical therapy for bone metastases^4–7^.

Tomasetti and Vogelstein recently described occurrence of random mutations during DNA replication in cancer stem cells playing an important role in the development of certain types of tumours^8^. This was associated with the development of cancer in which environmental or hereditable factors have a low impact on tumour onset and progression^9^, e.g. in prostate cancer. Developing new therapeutic strategies with high specificity for the malignant tissue is therefore of strong interest but challenging once prostate cancer progresses to an androgen-independent, hence hormone refractory state.

Aptamers are an emerging class of molecules for developing targeted therapy approaches^10,11^. They are single chained nucleic acids, folding into well-defined three-dimensional shapes based on which they recognise target structures with high affinity and specificity^12^. Aptamers targeting tumour cells are commonly identified by an *in vitro* selection process using cultured cell lines or isolated membrane proteins. However, only a few examples are described in which these aptamers are also capable of recognising the respective target or cells in the related *in vivo* microenvironment^13–19^. Recently, a prostate cancer targeting RNA aptamer selected in an internalisation cell-SELEX procedure was successfully used, in combination with two highly toxic drugs, for the inhibition of tumour growth *in vivo*^20^.

In contrast, selection procedures conducted solely *in vivo*, e.g. using xenograft tumour model systems are supposedly suitable for the *de novo* identification of effective tumour recognising aptamers. Here, an *in vivo* selection approach is described, employing DNA libraries for the identification of aptamers targeting androgen independent prostate tumours in an orthotopic xenograft mouse model. This approach resulted in the identification of a series of DNA aptamers that show tumour targeting properties in orthotopic and subcutaneous xenograft mouse models. Among them, one representative aptamer (D3P-21) was further characterised and found to reproducibly recognise prostate tumours *in vivo*. The interaction properties of D3P-21 were found to depend strictly upon the presence of a 5′-polyethylen glycol (PEG) moiety already implemented during the *in vivo* selection procedure. Spectroscopic data indicate that the conformation of the DNA aptamer is not impaired by the PEG moiety, hence it might directly interact with the target structures, explaining D3P-21’s PEG-dependent interaction properties.

The study opens the path towards *in vivo* selection procedures using DNA libraries in suitable model systems, an endeavour which previously has been supposed to be non-effective due to low stability and the rapid clearance of DNA *in vivo*. The data not only provide evidence for DNA aptamers being suitable for this approach but also that DNA aptamers are less immunogenic than previously anticipated^21^. Based on the study results, DNA aptamers may be revived as a less costly and alternative class of aptamers for basic research *in vivo* and therapeutic use. To overcome the reluctance of using DNA aptamers for *in vivo* purposes, more studies are required showing proof-of-concept and superior performance in validation studies. A systematic analysis of the potential of DNA aptamers to induce innate immune responses will be necessary, to address the probability of these compounds in a broad manner. Moreover, DNA is more robust and chemically stable than RNA and accepts chemical modifications as well as non-canonical nucleobases, which can be used for extending the genetic alphabet^22–25^.

## Results

**Figure 1:**
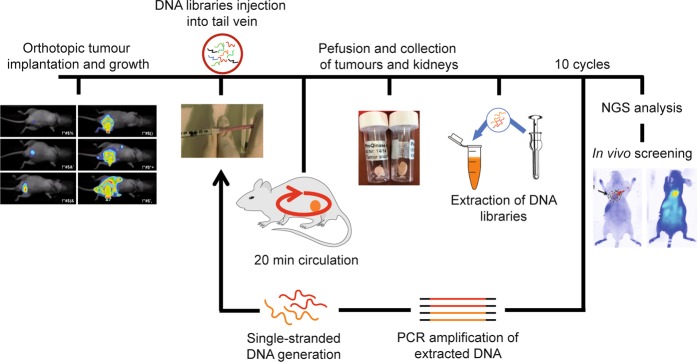
*In vivo* selection process using orthotopically xenograft prostate cancer models. PC-3 cells expressing luciferase were implanted to the prostate of nude mice and the tumour growth was monitored by bioluminescence imaging during 5 weeks until complete growth of the tumours. Pegylated (D3P) or naive DNA libraries (D3) were injected *via* the tail vein. After 20 min, mice were perfused and tumours and kidneys extracted and snap frozen. Homogenisation of the tumours and control organ, kidney, for extraction of bound oligonucleotides was then performed by means of mechanical and chemical homogenisation followed by phenol/chloroform purification and ethanol precipitation for D3 library and silica columns purification for D3P library. Extracted oligonucleotides were amplified and ssDNA was generated for the next SELEX cycle. In total, 10 selection cycles were performed for both libraries. NGS analysis of the *in vivo* SELEX was performed and selected identified sequences were tested by an *in vivo* screening assay in orthotopic and subcutaneous mouse models.

The general outline of the *in vivo* selection scheme using orthotopic xenograft prostate tumour models is depicted in Fig. 1. The prostate cancer cell line PC-3 was implanted into the prostate of male NMRI nude mice. PC-3 is a human hormone insensitive prostate tumour cell line, which represents a well-accepted tumour model for castration resistant prostate cancer^26^ with respect to its sensitivity to current treatments, *i*.*e*. exhibiting tumour growth inhibitory effects after treatment with docetaxel^27^ and its metastatic profile. The used cell line bears an intrinsic luciferase reporter protein enabling post-surgery monitoring of the tumour growth (Suppl. Fig. S1a). Approx. five weeks after implantation, the respective DNA library or phosphate buffered saline (PBS) was injected into the mice’s tail vein (Suppl. Fig. S1b). Tumours from mice injected with PBS were used as negative tumours during the library extraction and amplification steps to discard contamination of the samples (Supp. Fig. S2a,b).

Briefly, we established two *in viv*o selection protocols using the DNA library D3. The first selection protocol employed D3 in its naïve variant, whereas the second protocol made use of a 5′–polyethylene glycol (PEG, 11 kDa) modified version, named D3P. Besides the nature of the DNA library used, both protocols differed in the work up procedure of the DNA molecules after tumour and kidney resection and homogenisation (for details please refer to the methods section). The PEG moiety was chosen as it has been shown to increase the half-life of DNA molecules in the blood circulation^28,29^. Prior to injection, the DNA libraries were prepared as a solution in PBS. After 20 minutes of circulation, the mice were perfused with PBS and sacrificed by cervical dislocation. Subsequently, the prostate tumour was removed, snap frozen, and stored at −80 °C until further processing. After thawing, the tissues of three mice were homogenised as one sample and the nucleic acid library recovered either by silica column purification (D3P) or phenol/chloroform extraction followed by ethanol precipitation (D3). After recovery, the library was amplified by PCR, subjected to single-strand generation and used for the next selection cycle. In each selection cycle, the DNA molecules associated with the kidneys were also recovered and subjected to PCR, which allowed to control the general workflow as the kidneys represent the major clearance pathway of DNA aptamers *in vivo*^10^. As further control, we prepared tumours from mice injected with PBS only and subjected these tumours to the same recovery procedure and PCR protocol (Suppl. Fig. S2a,b). In Supplementary Table S1, the weights of the resected tumours (between 28 and 388 mg five weeks after implantation) and kidneys from each selection cycle are given. After ten *in vivo* selection cycles (the conditions of which are summarised in Suppl. Table S2), we analysed each of the obtained DNA libraries by next-generation sequencing (NGS)^30^. Between 1.5·10^5^ and 1.2·10^7^ sequences per selection cycle were analysed from both selection procedures (Suppl. Table S3).

**Figure 2:**
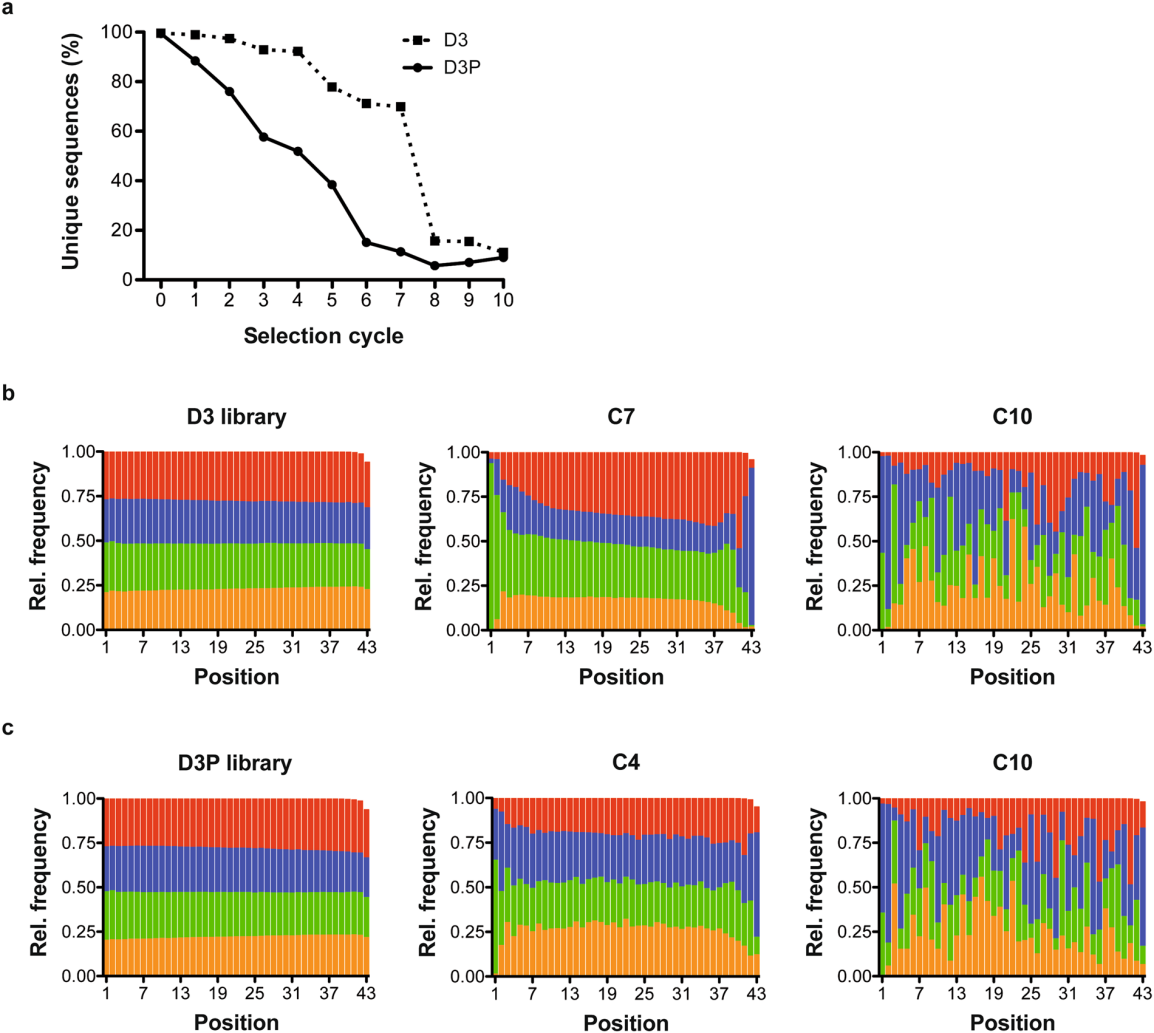
Next-generation sequence analysis of the DNA libraries D3 and D3P obtained by *in vivo* selection. (**a**) Analysis of the number of unique sequences within the obtained libraries from the *in vivo* selection using D3 (squares) and D3P (dots). The distribution of nucleotides over the 43 nucleobases of the initial random region of the starting library and the DNA libraries form the selection cycles 7 and 10 of the *in vivo* selection using D3 (**b**) and the distribution of nucleotides over the 43 nucleobases of the initial random region of the starting library and the DNA libraries from selection cycles 4 and 10 of the *in vivo* selection using D3P (**c**). Orange: dA, green: dC, blue: dG, and red: dT.

**Figure 3:**
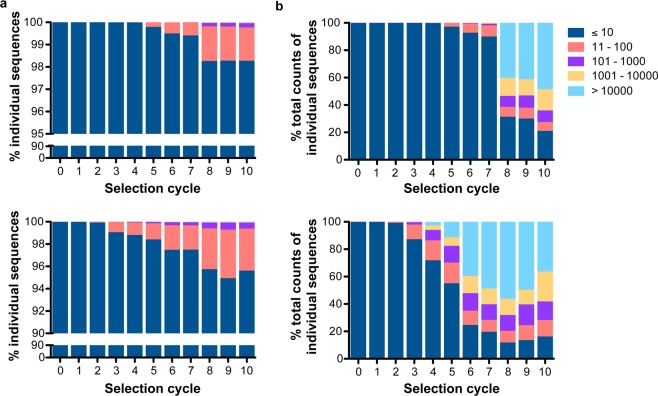
Next-generation sequence analysis of the DNA population obtained from sequencing the individual DNA libraries of the indicated *in vivo* selection cycles. (**a**) Individual sequences were grouped according to their frequency in the libraries derived from each selection cycle. Upper panel refers to the libraries obtained from the *in vivo* selection using D3; lower panel refers to the libraries obtained from the *in vivo* selection using D3P. (**b**) The proportion of those sequences in the total population. Upper panel refers to the libraries obtained from the *in vivo* selection using D3; lower panel refers to the libraries obtained from the *in vivo* selection using D3P.

The number of unique sequences was found to decrease significantly in later cycles, indicating both libraries being enriched (Fig. 2a). The PEG-modified DNA library D3P showed a steady decrease of unique sequences over the monitored DNA populations obtained from the different selection cycles. In contrast, the number of unique sequences in the naïve DNA library D3 was strongly decreased from the selection cycle 7 to 8 (Fig. 2a). This behaviour was also eminent from the distribution of the four nucleotides (dA, dG, dT, and dC) throughout the initial random region, which was similar up to selection cycle 4 and 7 of the libraries D3P or D3, respectively (Fig. 2b,c and Suppl. Fig. S3a,b). In turn, the nucleotide distribution was clearly altered when analysing the DNA populations obtained after ten selection cycles, which already became evident in selection cycles 6 (D3P) and 8 (D3) (Suppl. Fig. S3a,b). In the following and if not otherwise stated, we mainly describe the DNA populations of both libraries obtained after 10 selection cycles. Both libraries still contained unique sequences (11% (D3) and 9% (D3P)), which might be due to the high diversity of targets and the complex nature of the *in vivo* selection approach. Of note, 0.27% of the sequences were found to be present in the DNA populations of both libraries from the 10^th^ selection cycle, which can be explained due to i) common unspecific targeting, ii) higher resistance to nucleases or iii) a more favourable amplification by PCR. The sequences were grouped in different populations according to their copy numbers in each selection cycle, *i*.*e*. ≤10, 11–100, 101–1000, 1001–10000, and >10000 and normalised with respect to the total number of copies in each selection cycle (Fig. 3a,b). This grouping reveals that sequences with >10 copy numbers are increasingly observed from selection cycle 5 and 3 for D3 and D3P, respectively (Fig. 3a). However, the vast majority of sequences within the DNA population from these selection cycles have copy numbers of 10 or less (98.3% in D3 and 95.6% in D3P). In contrast at cycle 10, 48.5% of the sequences from the D3 library and 36.3% of those found in the D3P library correspond to few strongly enriched sequences (among them 25 (D3) and 7 (D3P) sequences) with >10000 copies (Fig. 3b).

Next, individual sequences were chosen for further testing based on their enrichment profiles. In particular, collection was defined by i) the frequency of an individual sequence in a DNA population and ii) amplification fold, *i*.*e*. change of copy numbers from one selection cycle to another (Suppl. Fig. S4a,b). Based on these criteria 46 sequences (22 from D3 and 24 from D3P) were selected for further assessment, among them 17 sequences with low copy numbers, *i*.*e*. frequency <0.5% but enrichment profiles similar to those found for the most abundant sequences (Suppl. Tables S4 and S5 and Suppl. Fig. S4a,b). The other 29 sequences were the most abundant sequences found with frequencies >0.5%, whereas one sequence, named either D3-0 (24.23%) or D3P-7 (5.63%), was found in both libraries. Of note is the frequency of each sequence found in the kidney. In contrast to the selection done with D3, where lower copy numbers of each individual sequence were found in the kidney compared to the tumour tissue, the selection using D3P revealed sequences with higher copy numbers associated with the kidney compared to the tumour (Supp. Table S4 and S5). This observation might be due to a more rapid renal clearance of the non-pegylated (D3) sequences (MW ~25 kDa, which is below the cut-off threshold of the renal glomerulus^31^) in comparison to the pegylated sequences (D3P, MW ~36 kDa).

**Figure 4:**
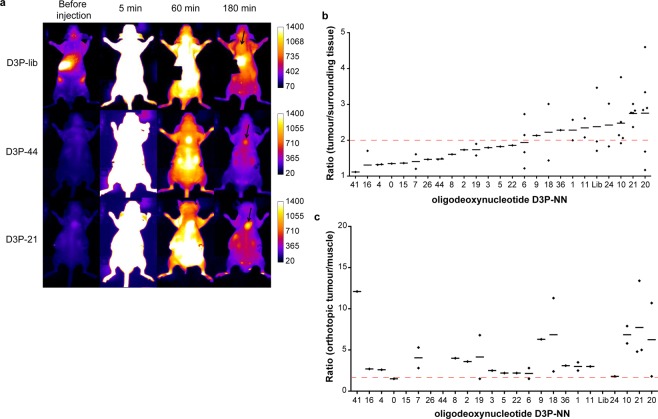
Planar imaging of pegylated sequences. (**a**) Imaging of mice injected with 2 nmol of the D3P-library, D3P-44, and D3P-21 at different time points post injection (0, 5, 60, and 180 min). Dorsal view; exposure time 1000 ms. Arrows depicted in mice images at 180 min point the subcutaneous tumour. (**b**) Ratio of subcutaneous fluorescence signal obtained from the tumour tissue compared to surrounding tissue (please see also Suppl. Fig. S5a, left panel) of all indicated sequences 180 min post injection. (**c**) Ratio of the fluorescence signal of the orthotopic tumour *ex vivo* compared to muscle fluorescence (please see also Suppl. Fig. S5a, right panel) of all indicated sequences at 180 min post injection. Five mice did not bear an orthotopic tumour, wherefore no values are given in (**c**) for the sequences D3P-15, D3P-26, D3P-44, and D3P-library.

We subjected all 46 sequences to an initial *in vivo* screening procedure using variants of the sequences bearing a near-infrared fluorescent dye (Alexa Fluor 680) at their 3′-ends. Sequences obtained from the library D3P were additionally equipped with a 5′–11 kDa PEG moiety. The sequences were evaluated using fluorescence reflectance imaging (FRI) of mice that bear orthotopic and subcutaneous xenograft tumours. The latter was necessary since the renal clearance of oligonucleotides hampers differentiation of sequences enriched in the orthotopic prostate tumours from those eliminated through the bladder. Two nanomole of the individual sequences were injected in the tail vein of anesthetised mice and whole-body FRI of the dorsal side view was performed before, 5, 60, and 180 minutes post injection. Subsequently, all animals were euthanised and several organs, including the orthotopic and the subcutaneous tumours, were harvested and analysed by e*x vivo* FRI. An initial screening using a single mouse per individual sequence was performed. Each sequence was evaluated for tumour targeting comparing the mean fluorescence inside the subcutaneous tumour to the mean fluorescence measured in a healthy zone adjacent to the tumour (Suppl. Fig. S5a). No clear differences were measured at 5 and 60 minutes after injection, due to a high background fluorescence before clearance of non-bound sequences, but several sequences provided a two-fold higher fluorescence signal in the tumour compared to the healthy zone after 180 minutes (Fig. 4a,b and Suppl. Fig. S5b). These sequences were subjected to further testing, which included the sequences D3P-16, –6, and –7 that revealed a lower fluorescence ratio of the tumour *vs*. the normal tissue and considered as non- or weak-targeting sequences (Fig. 4b and Suppl. Fig. S5b). Of note, none of the sequences derived from the library D3 were found to target efficiently the subcutaneous tumours (Suppl. Figs S5c and S6).

**Figure 5:**
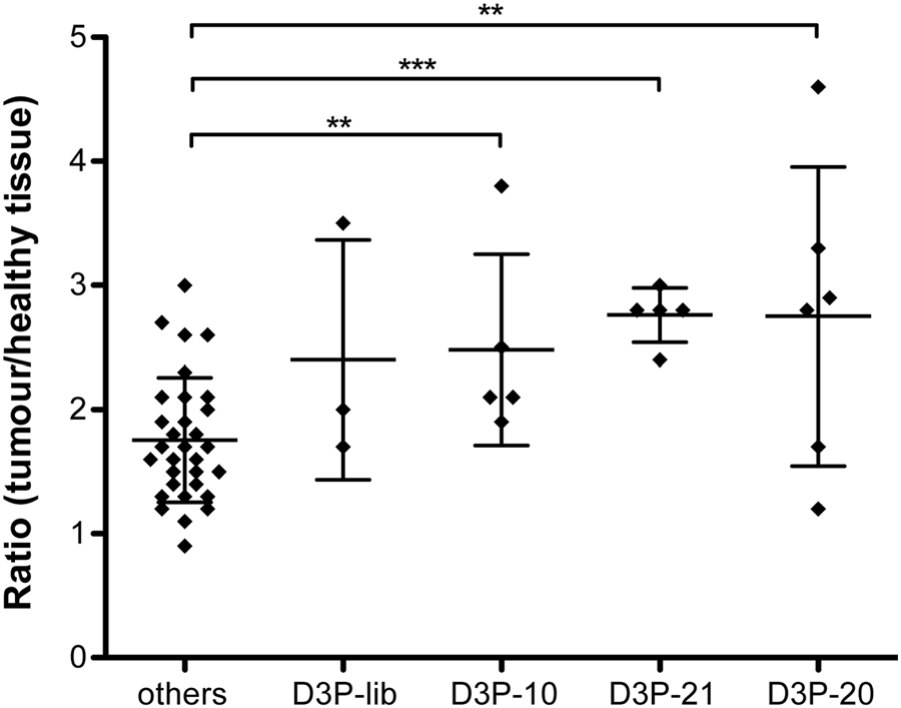
Validation of aptamers evolved from the DNA library D3P by planar imaging. The ratio of subcutaneous fluorescence signal obtained from the tumour tissue compared to surrounding tissue (please see also Suppl. Fig. S5a, left panel) of all indicated sequences 180 min post injection is given. The values obtained from the starting library (D3P-lib) (n = 3), and the aptamers D3P-10 (n = 5), D3P-20 (n = 6), as well as D3P-21 (n = 5) were compared to all other sequences tested and pooled as “others” (n = 31). The aptamers D3P-21, D3P-P20 and D3P-P10 have a statistically significantly higher tumour to healthy tissue ratios (2.76 ± 0.09, 2.75 ± 0.49, and 2.48 ± 0.34, respectively) compared to the mice injected with the other aptamers (1.75 ± 0.09). In contrast, the difference obtained with the naïve library (D3P-lib) (2.400 ± 0.55) was not significant. Statistical significance was calculated using Graphpad Prism 6 using an unpaired t test model assuming that all data have the same standard deviation (SD). **P < 0.01 and ***P < 0.001.

Furthermore, the calculated ratios obtained by FRI were ≤2 for most of the sequences (Suppl. Fig. S6). The repetition of the screening experiments with the sequences D3-39, -21, -34, and -27 revealed a high variation of obtained ratios and in some cases (D3-39 and D3-21) a strong fluorescence signal detectable throughout the whole mouse. Due to these findings, the D3-related sequences were decided not being further evaluated. Instead we focussed on the characterisation of the sequences obtained from the selection experiments using D3P. The majority of re-screened sequences obtained from the library D3P also showed a high variation and difference in their biodistribution, e.g. D3P-1, -4, -6, -7, -11, -16, -18, -19, -24, and -44 (Fig. 4b and Suppl. Fig. S5b). Orthotopic tumour targeting was also assessed by *ex vivo* fluorescence measurements comparing the fluorescence of prostatic tumours *vs*. muscle. However, this analysis was limited since half of the mice used during our screening did not develop orthotopic tumours in the prostate. Nevertheless, the fluorescence ratio between prostate tumour and muscle were ≥2 for most of the sequences tested and even higher ratios were obtained by D3P-10 and D3P-21 (Fig. 4c). Together with D3P-20, these two sequences revealed superior subcutaneous tumour targeting as well (Fig. 4b). Further testing of these sequences using additional subcutaneous mouse model samples revealed a more reproducible tumour targeting when compared to the starting library and all other analysed sequences (Fig. 5). Among them, D3P-21 was the most promising sequence as it showed an average fluorescence ratio of tumour to healthy tissue of 2.76 ± 0.09, which is higher and more reproducible compared to the average ratio obtained by all other sequences (1.75 ± 0.09).

For further validation, the amount of aptamer localised to the tumour tissue was quantified by quantitative PCR (qPCR). Both orthotopic and subcutaneous tumours from mice treated with D3P-21 or the D3P-library were homogenised and the DNA extracted. The obtained DNA was then subjected to qPCR (Suppl. Fig. S7a). This analysis revealed a higher amount of D3P-21 recovered from subcutaneous tumours compared to the D3P-library (Suppl. Fig. S7a left). Interestingly, qPCR data also reveal that more copies of D3P-21 could be recovered from the orthotopic compared to the subcutaneous prostate tumour tissue (Suppl. Fig. S7a, right). The heterogeneity of the recovered DNA amounts might be explained by the fact that the samples of the test set were non-perfused (in contrast to the samples directly obtained from the selection procedures and which were used as sources to generate the results shown in in Figs 2, 3 and Supp. Figs S2–S4), resulting in residual amounts of blood remaining in the tissue that interfere in the analysis.

A pre-requisite for the potential therapeutic application of aptamers *in vivo* is their long-term stability in mammalian serum. As D3P-21 was selected *in vivo*, this inherently indicates that the sequence has certain nuclease resistance. To further explore its stability, its degradation in serum (Suppl. Fig. S7b, quadrants) and in Dulbeccos’s phosphate buffer saline (DPBS) (Suppl. Fig. S7b, circles) as a control were tested. These experiments revealed that after 1 h of incubation at 37 °C, 92.8% of the aptamer D3P-21 remain full length (Suppl. Fig. S7b, left panel) in serum, which is ~20% more compared to its non-pegylated variant (73.1%) (Suppl. Fig. S7b, right panel). This difference becomes less significant after 3 hours of incubation, after which 61.1% (D3P-21) and 50.8% (non-pegylated D3P-21) of the respective full-length aptamers were detected. These data are in line with the qPCR results that also project towards a good stability of D3P-21 *in vivo*. Of note, while the PEG moiety seems to increase the endurance of D3P-21 towards nucleases up to 3 h, extended incubation times revealed even more degradation of the pegylated variant compared to the non-pegylated aptamer. When incubated in DPBS, no degradation of the aptamer variants was observed.

**Figure 6:**
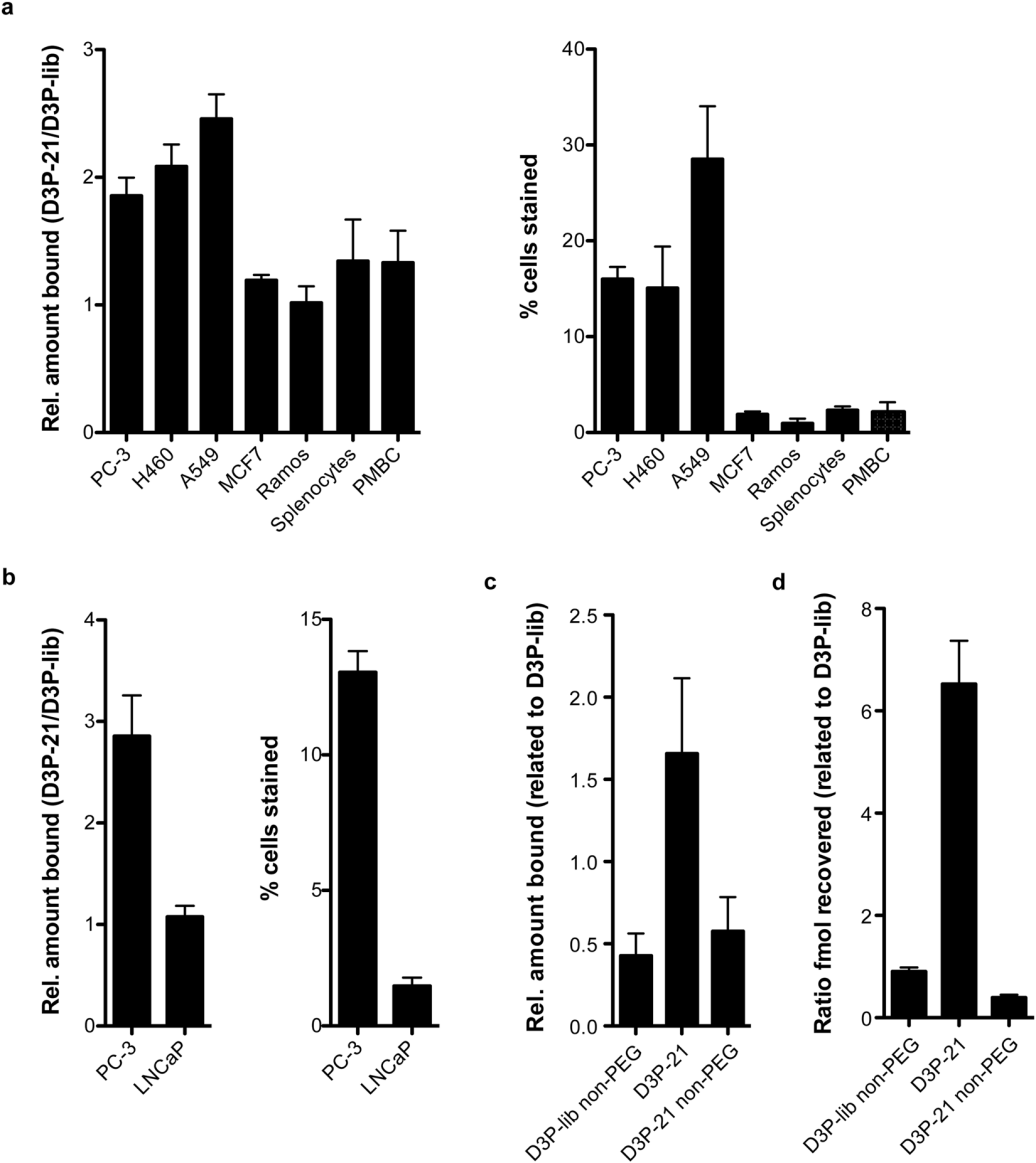
Evaluation of D3P-21 properties *in vitro*. (**a**) Flow cytometry analysis of the interaction of aptamer D3P-21 and the DNA library D3P (D3P-lib) with prostate cancer PC-3 cells, murine splenocytes, and murine peripheral blood mononuclear cells (PBMC) and other cancer cell lines (MCF7, H460, A549, and Ramos). Depicted is the ratio of binding of D3P-21 compared to the D3P-library (left panel) and the total percentage of cells bound by D3P-21 in (right panel). Cells were incubated with 200 nM of the D3P-21 or D3P-lib labelled at the 3′-end with Alexa Fluor 680. (**b**) Flow cytometry analysis of the interaction of D3P-21 and D3P-lib with the prostate cancer PC-3 and LNCaP cells. Shown is the ratio of binding of D3P-21 compared to D3P-lib (left panel) and the total percentage of cells bound by D3P-21 (right panel). Cells were incubated with 100 nM of the aptamer or D3P-lib labelled with Atto647N at the 3′-end. (**c**) Flow cytometry analysis of the impact of the PEG moiety of D3P-21 on its interaction properties. PC-3 cells were incubated with pegylated (D3P-21), non-pegylated D3P-21 (D3P-21non-PEG), or the non-pegylated D3P-library (D3P-lib non-PEG) labelled with Atto647N at the 3′-end and the ratio of binding is shown compared to D3P-lib. (**d**) Analysis of the impact of the PEG moiety on D3P-21 on PC-3 cell interaction using non-labelled DNA and qPCR for quantification. Shown is the ratio of recovered fmoles of the indicated oligodeoxynucelotide compared to D3P-lib. Represented as mean ± SD (n = 4).

After having identified the aptamer D3P-21 and validated its performance *in vivo*, the aptamer’s characteristics and properties *in vitro* were analysed. To characterise the binding properties of D3P-21 *in vitro*, flow cytometry studies were performed using PC-3 cells. In these experiments, the aptamer D3P-21 revealed a two-fold increase in binding to cultured PC-3 cells compared to the naïve D3P library (Fig. 6a and Suppl. Fig. S8). Subsequently, the interaction of D3P-21 with other cancer cell lines, *i*.*e*. A459, H460, MCF-7, Ramos, and the androgen-dependent prostate cancer cell line LNCaP was tested. Furthermore, peripheral blood mononuclear cell (PMBC) and splenocytes from mice were also investigated. Of note, for technical reasons oligonucleotides (ODN, aptamer and controls) labelled with Atto647N fluorophore were used in the flow cytometry assay with LNCaP cells, while ODNs (aptamer and controls) labelled with Alexa Fluor 680 were employed for the other cell lines. These experiments revealed that D3P-21 interacts with the lung cancer cell lines A549 and H460 (lung carcinoma), while no binding to splenocytes, PMBCs, Ramos (Burkitt’s lymphoma), and MCF7 (breast cancer) cells was observed (Fig. 6a and Suppl. Fig. S8). Notably, binding to androgen-dependent prostate cancer LNCaP cells was also not observed (Fig. 6b and Suppl. Fig. S9). Other aptamer candidates tested (D3P-20, D3P-10, D3-28, D3-39, and D3-N1) showed no binding to PC-3 cells *in vitro* (Suppl. Figs S10a,b, S11a,b).

Next, the impact of the PEG moiety on the binding characteristics of D3P-21 was analysed. Therefore, the interaction of pegylated or non-pegylated D3P-21 with PC-3 cells was measured by flow cytometry (Fig. 6c and Suppl. Fig. S12) and qPCR (Fig. 6d) and compared to the D3 and D3P libraries, respectively. These experiments demonstrated a loss of binding performance of D3P-21 in the absence of the PEG moiety. Conversely, the binding of the D3 library to PC-3 cells was found to be independent of its pegylation status (Fig. 6c,d).

**Figure 7:**
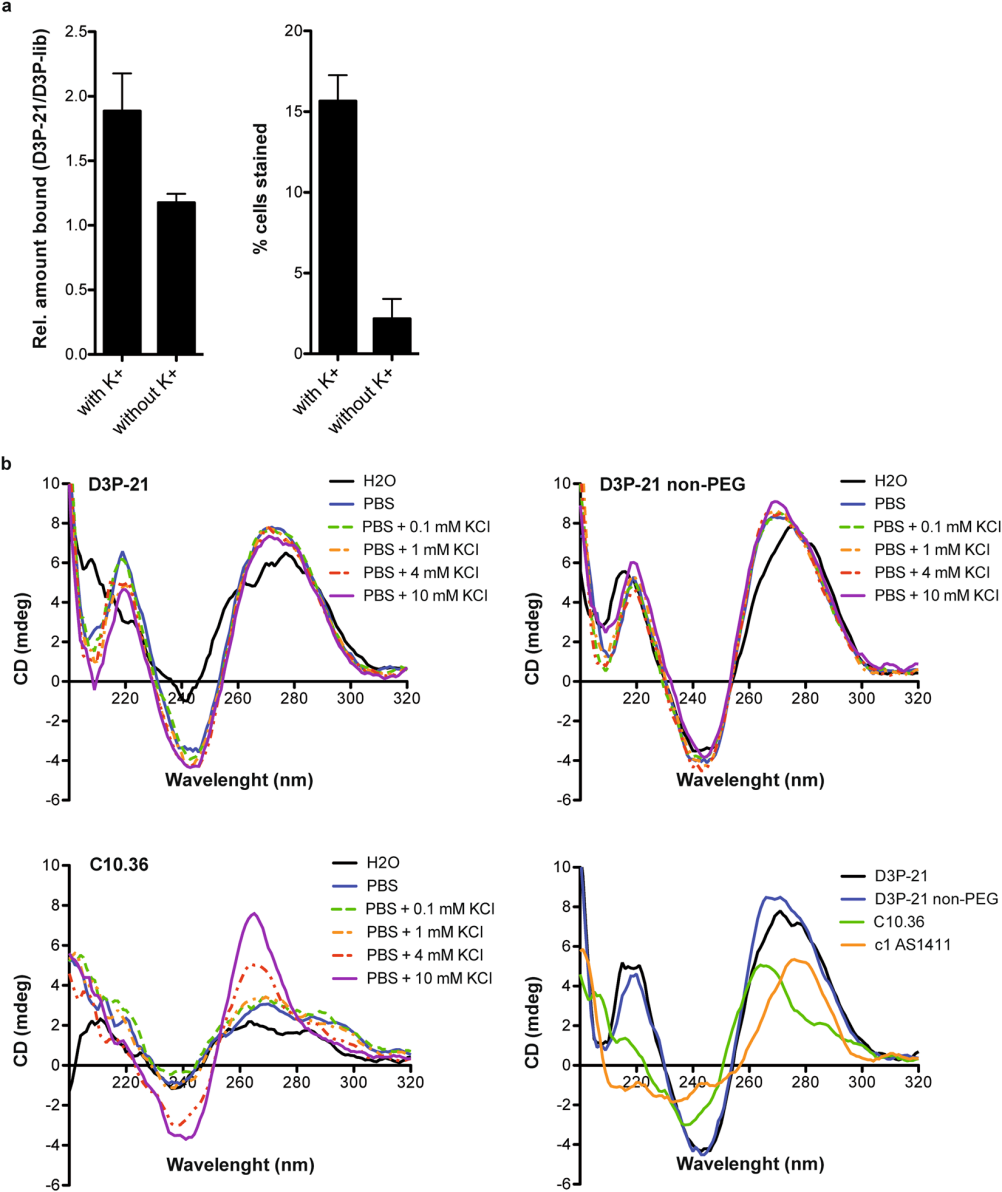
Impact of potassium ions on the interaction D3P-21 with PC-3 cells. (**a**) Flow cytometry analysis to monitor the influence of potassium in the binding of aptamer D3P-21 to PC-3 cells. Shown is the ratio of binding of D3P-21 compared to D3P-lib (left panel) and the total percent of cells bound by D3P-21 (right panel). The cells were incubated with 100 nM of the aptamer labelled with Atto647N at the 3′-end. Represented as mean ± SD (n = 4). (**b**) CD spectroscopy analysis of D3P-21. CD spectra of D3P-21 (upper left panel), non-pegylated D3P-21 (D3P-21 non-PEG) (upper right panel), and C10.36 (lower left panel) under different conditions, *i*.*e*. H_2_O, PBS w/o potassium ions (pH 7.4), and PBS with increasing concentrations of potassium ions as indicated. Comparison of the obtained CD spectra of different aptamers and variants at a concentration of 4 mM potassium ions (lower right panel).

D3P-21 is a G-rich sequence (33.75%, Suppl. Table S5) and, thus, might be capable of folding into G-quadruplex structures^32^. We therefore analysed the dependence of cell binding of the aptamer on the presence of potassium ions as these are key to G-quadruplex structure stabilisation we analysed the dependence of cell binding of the aptamer on the presence of potassium ions as these are key to G-quadruplex structure stabilisation^33^. The obtained results show that the interaction of D3-P21 with PC-3 cells depends on the presence of potassium ions in the binding buffer (Fig. 7a and Supp. Fig. 13). In order to elucidate whether D3P-21 forms a G-quadruplex structure and to analyse the impact of the PEG moiety on the conformation of D3P-21, circular dichroism (CD) spectroscopy experiments were performed. CD spectra of the aptamer were characterised by a positive peak at 273 nm (D3P-21) and 270 nm (non-pegylated D3P-21) and a negative peak at 244 nm (Fig. 7b, upper left (D3P-21) and right (D3P-21 non-PEG) panels). These typical CD profiles indicate that D3P-21 most likely contains a B-form conformation^34^. Notably, the absence or presence of potassium ions did not have an impact on the CD spectra, indicating that the conformation of D3P-21 is K^+^-independent. In contrast, the CD spectra of C10.36, a previously described parallel G-quadruplex containing aptamer^35^, were found to be strongly affected by the absence or presence of K^+^ (Fig. 7b, lower left panel). Minor differences were observed in the CD spectra of the pegylated *vs*. the non-pegylated aptamer revealing that the 11 kDa PEG moiety has little or no effect on the aptamer’s conformation (Fig. 7b, lower right panel). These data also indicate, that D3P-21 most likely does not fold into a common G-quadruplex structure.

**Figure 8:**
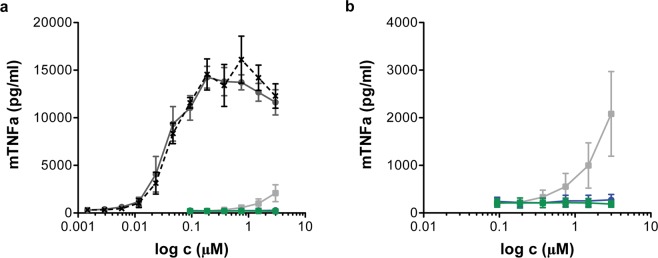
Evaluation of aptamer immunogenicity. (**a**) Increasing concentrations of D3P-library (grey line), D3P-21 (blue), D3P-21 non-pegylated (green), CpG ODN 1826 type B (dark grey) and LPS (black) were incubated with immortalized murine embryonic stem cell-derived macrophages for 24 h and concentration of TNF-alpha in the supernatant was determined by HTRF assay. Results without CpG ODN and LPS are depicted in (**b**). Represented as mean ± SEM (n = 5).

Finally, the impact of D3P-21 on the innate immune system was evaluated since previous studies suggest that DNA aptamers activate the immune system, mainly mediated by the Toll-like receptor (TLR) superfamily^21^. To determine D3P-21’s potential in this regard, the secretion of TNFα by immortalised murine embryonic stem cell-derived macrophages upon aptamer treatment was measured^36^. The results demonstrated a very low immunogenicity by D3P-21 and its non-pegylated variant at concentrations up to 3 µM (Fig. 8a,b). In contrast, the D3P-library induced TNFα secretion at concentrations above 0.375 µM. This finding is not surprising as the initial library contains ~10^15^ sequences, some of which most likely sharing structural motifs capable of TLR recognition. However, the activation of the innate immune response by the DNA library was found to be >3 orders of magnitude less pronounced compared to controls, *i*.*e*. CpG and LPS^37^. These data indicate that D3P-21 does not activate the innate immune system.

## Discussion

Nuclease-mediated degradation and rapid renal filtration of nucleic acids categorises the *in vivo* selection of aptamers highly challenging. To date, a few examples of *in vivo* selected aptamers are described employing 2′-deoxy-2′-fluoro pyrimidine (2′-fluoro)-modified RNA molecules^38–40^ and more recently, with a phosphorothioate containing DNA library^41^. 2′-fluoro RNA is considered to be more resistant toward serum nucleases and less prone to elicit innate immune responses^31^. Likewise, the majority of aptamers investigated for therapeutic approaches are built from 2′-fluoro RNA^42–44^. Exceptions are thrombin inhibiting aptamers and AS1411, all of which were shown to fold into highly stable G-quadruplex structures. These G-quadruplexes are investigated *in vivo* for the treatment of coronary artery syndrome or acute myeloid leukaemia^45–47^. In comparison to DNA molecules, 2′-fluoro RNA holds a series of disadvantages, *i*.*e*. very cost demanding and, thus, more difficult to engineer and optimise. In addition, 2′-fluoro RNA aptamers extending 50 nucleotides in length are still difficult to synthesise chemically and in larger quantities, as required for therapeutic approaches^10^. Thus, a minimisation of the active aptamer might be required before subjection to further therapeutic development. These characteristics make 2′-fluoro RNA challenging to be employed for basic research and drug development. Alternatively, DNA is affordable but branded as unstable and to evoke immune responses, e.g. mediated by the Toll-like receptor superfamily^21^. To this end, neither a systemic investigation of the inherent capability of DNA aptamers to activate the innate immune system nor their eligibility for *in vivo* selection approaches has been studied comprehensively.

Herein, an *in vivo* selection procedure is described that enabled the identification of prostate tumour recognising DNA aptamers. An orthotopic xenograft mouse model was applied during the selection and validation procedure to secure tissue penetration and recognition of the relevant microenvironment of the tumour by the resultant aptamers. Orthotopically implanted xenograft tumour models are considered to represent one of the most accurate and available mimics of the tumour microenvironment in regard to a patient’s clinical situation. The androgen-independent PC-3 cell line was used for implantation, which is hormone independent and PSA negative. Hormone refractory prostate tumours are considered being non-curable yet^26^.

The overall results of the *in vivo* selection experiments and the fact that the best performing aptamer, D3P-21, was enriched from a pegylated DNA library indicate that such libraries reveal superior performance compared to a naïve DNA library. The tested sequences obtained from the selection using the naïve DNA library did not reveal a consistently performing aptamer yet. However, it cannot be excluded that a well-performing aptamer could still be identified from this library as the enriched diverse population may contain these sequences albeit at very low abundance and the tools yet applied to analyze the library are not sufficient to reveal them. The continuation of the *in vivo* selection process might further enrich targeting sequences from this library as well. Likewise, D3P-21 was ranked on position 17 of all identified sequences regarding copy numbers and frequency within the enriched pegylated DNA library. Noteworthy, the most abundant sequences were not found to be the best targeting aptamers. This observation can be explained by a higher circulation half-life, targeting of non-tumour related structures, or by being more prone to PCR replication and thereby biasing the sequence populations^48^.

A rapid renal clearance of the naïve DNA library (D3) compared to a PEG-modified variant might also account for unsuccessful use during the *in vivo* selection approach. Renal clearance is due to the average molecular weight of D3 of ~25 kDa, which is below the cut-off threshold of the renal glomerulus^31^. In turn, an increased circulation time of the PEG-modified library might enhance the exposure time of candidate sequences to the relevant tumour tissue. Thus, under this scenario the enrichment of specifically associated aptamers is favoured compared to enriching for those sequences with simply higher resistance towards nuclease degradation, e.g. because of appropriate structure formation. This effect was intended by the addition of the 11 kDa PEG moiety to the D3P-library, thereby increasing the library’s molecular weight to ~ 36 kDa, which is in the range of the renal glomerulus threshold^31^. It has also been demonstrated that pegylation could induce steric repulsion of plasma proteins and decrease phagocytosis by the reticuloendothelial system^49^. It is unknown whether even larger PEG moieties would improve the selection outcome, but as PEG has recently been linked with side effects, *i*.*e*. allergy responses, anaphylactic reactions, and hypersensitivity in humans^50–52^, alternative tags such as lipids or protein carriers might also be worthwhile to investigate in the context of *in vivo* selection experiments^53,54^.

D3P-21 showed consistent tumour targeting in the employed orthotopic and subcutaneous xenograft mouse models. The aptamer followed a constant enrichment profile, with steadily increasing frequency up to 0.94% in the DNA population obtained after 10 selection cycles. The maximal fold of amplification was seen from selection cycle 3 to 4, while it was predominantly from cycle 2 to 3 for most of the highly abundant sequences. This finding underlines the importance of considering less frequent sequences for further characterisation instead of focussing only on the most abundant ones, as similarly described in previous literature reports^55,56^. Sophisticated population analysis procedures are of particular importance for aptamer selection approaches using complex target structures, e.g. cells, exosomes^57^, and tissues^58^, albeit still in its infancy but thought to develop rapidly alongside the availability and analysis of next-generation sequencing data^59,60^. The NGS-analysis further revealed that the sequence population from the last two selection cycles did not change dramatically, e.g. no new enriched sequences were detected within these populations. The data shown here underline NGS being an indispensable tool to track the evolution of DNA populations during *in vivo* selection procedures and for the identification of potential candidate aptamers. It might be supportive in detecting aptamers already in early selection cycles, thus reducing the entire number of selection cycles, which is of great importance when costly and time-consuming experiments as well as living animals are required. This study provides a comprehensive and unprecedented population analysis of the progression of an *in vivo* selection process of DNA aptamers by NGS. In contrast, the few reported *in vivo* selection procedures relied on standard cloning and Sanger sequencing^38,40^ of ~50 sequences for identifying aptamer candidates and only one study performed Sanger sequencing in comparison with deep sequencing^39^. However, owing to the nature of Sanger sequencing this procedure most probably reveals the most abundant sequences, which as implied by this study might limit the success rate.

Strong heterogeneity between different mice treated with the same candidate aptamer was observed for many of the tested sequences. This reflects the challenge of an *in vivo* screening approach, which was manifested also by different degrees of metastasis, *i*.*e*. in the bones, pancreas, liver, or abdomen between mice bearing orthotopic xenograft prostate tumours potentially affecting the biodistribution of aptamers. Nevertheless, aptamer D3P-21 revealed very low heterogeneity *in vivo*, when targeting the subcutaneous and orthotopic tumours.

D3P-21 also interacts with cultured PC-3 cells *in vitro*, which in turn demonstrates the capacity of this aptamer to recognise a target that is present on the tumour cells in culture and in the tumour microenvironment *in vivo*. Besides PC-3 cells, D3P-21 also recognises lung cancer cell lines, e.g. A549 and H460, whereas the breast cancer cell line MCF-7 and the androgen receptor and PSA positive prostate cancer cell line LNCaP were not bound. This finding is in line with recent data on mRNA expression profiles of various cell lines that reveal A459 cells clustering with PC-3 but not MCF-7^61^. This behaviour of D3P-21 is in accordance with previous studies that show aptamers selected by cell-SELEX recognising other cell lines than the one employed for the selection itself^18^ and specificity for a defined tumour tissue was not part of the *in vivo* selection regimen. No interaction of D3P-21 with murine splenocytes, PBMC’s, and human blood cancer cell lines, e.g. Ramos (Burkitt’s lymphoma), was detected. These data are in accordance with the *in vivo* selection setting, during which the DNA libraries when injected into the tail vein were ‘co-incubated’ with PBMCs, which most likely served as a parallel negative selection as only those DNA sequences associated with the tumour tissue were processed further. Together, these data indicate that D3P-21 is a tumour targeting aptamer with good specificity.

The aptamer’s cell binding properties depend on the presence of the PEG moiety and potassium ions. CD spectroscopy indicated that D3P-21 partially folds into a B-form helical structure but does not reveal a G-quadruplex. As the PEG moiety seems not to have a great impact on the aptamer’s folding it most likely directly interacts with the target structure of the cell surface. To the best of our knowledge, this is the first study demonstrating the successful selection of aptamers from a pegylated DNA library. The introduction of flanking moieties during selection has been proven successful compared to post-selection incorporation to avoid aptamer folding and binding issues^62^. Several literature examples are known in which the PEG was introduced post-selection, e.g. to improve the pharmacokinetic properties of aptamers^42,63,64^. The introduction of a PEG moiety in these aptamers did not affect their affinity to its target molecules significantly, however minor differences in binding were observed depending on the nature (linear or branched) and size of the PEG moiety^65^.

Targeting of tumour tissue in its native environment is a major challenge in cancer therapy in general and in particular for respective organs that are difficult to treat, e.g. prostate and pancreas. Aptamers represent an excellent compound class for achieving this. They can be selected *in vivo*, thereby optimally adapted towards their desired application^38–40^. Likewise, aptamers can also be selected *in vitro*, e.g. targeting cultured tumour cells or homogenously purified extracellular domains of proteins normally found on tumour cell surfaces^66^. The latter is greatly represented by a 2′-fluoro RNA aptamer that binds to prostate-specific membrane antigen (PSMA) and which has been shown to be valuable for targeting therapies in mice^63,67–70^. Which of the strategies of aptamer generation, *i*.*e*. *in vivo* and/or *in vitro* will be more straight forward in identifying aptamers with efficient targeting properties in a clinical setting though remains elusive yet.

In conclusion, the data in this study show that murine orthotopic xenograft models are compatible with *in vivo* selection experiments. As these model systems reflect the natural microenvironment of tumours in cancer biology very well, the resultant aptamers may bear great potential to be further developed as targeted therapeutic regimen. This finding also opens the path towards many more applicable orthotopic model systems, e.g. melanoma, breast cancer, colon cancer, pancreatic cancer, non-small cell lung cancer, and even metastasis model systems^71–74^, with which the developed procedure is compatible. The obtained data essentially illustrate the benefit of combining *in vivo* selection with high-throughput sequencing and thorough data analysis. The more sequencing data on further *in vivo* selection studies will be available the more knowledge on the evolution of sequences will be gained, which will support to make more direct hits on aptamers from these endeavours. We performed a comparative *in vivo* study, head-to-head evaluating pegylated *vs*. non-pegylated DNA libraries and conclude that the presence of PEG entities enhances selection success. Finally, the study establishes DNA molecules and libraries for *in vivo* selection procedures and might revive the application of DNA aptamers, besides 2′–fluoro RNA aptamers and beyond highly stable G-quadruplex structures as applicable compound class for developing targeted therapies and/or for validating targeting strategies. The recently demonstrated therapeutic efficacy of chemically modified DNA aptamers, so-called SOMAmers^75–77^ and the lack of stimulating the innate immune system by D3P-21, a major drawback traditionally linked to DNA, further supports this notion. In particular, future work will be required to reveal the molecular target of D3P-21, e.g., using pull-down experiments from the PC3 cell line followed by LC-MS/MS analysis, the dependence on the PEG moiety, and whether the aptamer functions as vehicle for the specific delivery of cargo therapeutics, e.g. paclitaxel or antagomirs to prostate tumours.

## Material and Methods

### Ethics Statement

All animal use procedures for subcutaneous PC-3 tumour models and *in vivo* fluorescence imaging were in strict accordance with the recommendations of the European Community (86/609/CEE) and the French National Committee (décret 87/848) for the care and use of laboratory animals. Ethics committee of CETEA – CEA DSV (Comité d’Ethique en Expérimentation Animale (CETEA), de la Direction des Sciences du Vivant (DSV) du Commissariat à l’Energie Atomique et aux énergies alternatives (CEA)) approved the study (ref: 12-093).

Experimental protocols for performing *in vivo* orthotopic PC-3 models and Aptamer *in vivo* selection studies had been approved by the Ethics Committee for Animal Experimentation, and were registered by the Regierungspräsidium Freiburg (G-12/62), Germany. The animal experimentation is in accordance with the European Community guideline 2010/63/EU.

### Orthotopic PC-3 tumour model

On Day 0.3·10^6^ PC-3 tumour cells expressing luciferase in 15 µl PBS were implanted orthotopically into 20 male NMRI nude mice. To prevent pain, Meloxicam (Metacam, 1 mg/kg, s.c.) was applied 1 h prior to surgery and 24 h post implantation. Male NMRI nude mice were anesthetised in a separate box using 1.5–2 Vol% Isoflurane with an oxygen flow of 0.6 l/min. The mice were positioned on a heated operating table with the left side upwards. The skin was cleaned, shaved and sterilised. An incision of approx. 1 cm was made in order to display the seminal vesicle and the prostate. A cell suspension of 3·10^6^ PC-3Luc cells in 15 µl PBS was injected orthotopically into the prostate using a 29 G needle syringe. The seminal vesicle and the prostate were carefully pushed back into the visceral cavity and the abdominal wall closed by suturation. Thereafter the mouse was warmed in a separate box while recovering from anaesthesia. In the following, animal weights were measured three times weekly (Monday, Wednesday and Friday).

### Subcutaneous PC-3 tumour model

For *in vivo* screening experiments, mice were subcutaneously injected between shoulder blades with 3·10^6^ PC-3 cells in a volume of 200 ml of Matrigel (BD Bioscience, Le Pont de Claix, France) and phosphate- buffered saline (PBS) (50:50). Tumours were then allowed to grow for 3–5 weeks until a size around 300 mm3 before *in vivo* imaging experiments. During each injection and imaging experiments, mice were anesthetised with isoflurane–1.25% in a 1:3 mixture of O2 and air. Subcutaneous injection was performed 3 weeks after orthotopical implantation as previously described or in mice without orthotopic implantation.

### *In vivo* bioluminescence imaging

During the course of the study, tumour growth was monitored *in vivo* using bioluminescence imaging. For this purpose, 150 mg/kg D-Luciferin was injected intraperitoneally (i.p.) into the mice 7 min before anesthetisation. Light emission was measured 10 min post injection with a CCD-camera for 5 min using a NightOWL LB 981 bioluminescence imaging system (Berthold Technologies, Germany).

### *In vivo* SELEX

All oligonucleotides, including DNA libraries and primers, were synthesised by Ella Biotech GmbH (Munich, Germany). Two separate 80-nt single-stranded DNA libraries, D3 and D3P, were used consisting in a 43-nt random region. D3P library contained an 11 kDa polyethylene glycol (PEG) moiety on the 5′-end. Both libraries were amplified by PCR using the following primers: forward primer (Fw) 5′-GCTGTGTGACTCCTGCAA-3′, with 5′–11kDa PEG moiety in the case of D3P library, and reverse primer (Rv-Pho) 5′-Phosphate-GGAGACAAGATACAGCTGC-3′). PCR reaction was performed by using GoTaq® G2 Flexi DNA Polymerase (Promega) and 1 µM of both Fw and Rv-Pho primers with the following cycling program (2 min 95 °C; 30 sec. 95 °C, 30 sec. 64 °C, 45 sec. 72 °C; hold 10 °C) in a Veriti 96 well thermal cycler (Applied Biosystems).

*In vivo* selection was done using tumour-bearing animals. Mice were either treated intravenously with one of two aptamer libraries (D3 or D3P) or left untreated. Five nmol of libraries (consisting of one copy of each sequence) were injected in cycle 1 and the amount was reduced to 2, 1 and 0.5 nmol for cycles 2, 3 and 4 respectively. From cycle 5 to 10, 0.1 nmol of the libraries were injected. Prior injection, libraries were prepared in 110 µL of Dulbecco’s phosphate-buffered saline (DPBS) containing Ca^++^ and Mg^++^ (Gibco), denatured at 80 °C for 3 min and slowly cooled down to RT for proper folding. After 20 min, animals were perfused with DPBS, killed by cervical dislocation and tumours and kidney were collected. Tumours and kidney were snap-frozen in liquid nitrogen and stored at −80 °C before recovering the nucleic acids from the tissues. Weight of the tumours and kidneys used during *in vivo* SELEX are summarised in Supp. Table S1. Extraction of the nucleic acids from 3 tumours and 3 kidneys from injected mice was performed by first homogenising the organs with a 7 mL dounce tissue grinder with large and small clearance pistils (Landgraf Laborsysteme HLL GmbH). To lyse the cells, TE-SDS Lysis buffer (0.1 M Tris-HCl pH 8, 1 mM EDTA pH 8, 0.5% SDS) supplemented with 0.5 µg/µL of Proteinase K (Roth) was used for D3 injected tumours or kidney, followed by 10 min incubation at 95 °C. For D3P library, buffers A1, A2 and A3 from NucleoSpin® Plasmid kit (Macherey-Nagel) were used for lysis of the cells followed by 10 min centrifugation at 4000 rcf to remove cellular debris. Purification of extracted oligonucleotides was performed by means of phenol/chloroform extraction and ethanol precipitation for D3 library, and by silica columns (DNA Clean & Concentrator^TM^ −500 (Zymo Research)) in the case of D3P library. A negative control was always included with tumours extracted from control mice (injected with DPBS) following the same procedure described above. Purified oligonucleotides were then re-dissolved in milliQ water and a first PCR amplification was performed. RNA digestion was performed before PCR amplification by using a 1:1 mixture of RNase T1 (Roche) and RNase A (Macharey-Nagel). Agarose gel (4%) purification was then performed in order to separate the library band from genomic DNA and primers with the NucleoSpin® Clean-Up kit. Further PCR amplification was then performed to reach the required amount of library the next cycle. All tissue homogenisations and PCR preparations were performed in two different PCR workstations (Peqlab) in order to avoid contaminations. Single strand displacement of the purified PCR product was carried out by λ-exonuclease digestion in 1 × λ-exonuclease reaction buffer and 5000 U/mL of λ-exonuclease (Thermo Scientific). After 30 min incubation at 37 °C, λ-exonuclease was inactivated at 80 °C for 10 min. Subsequently the samples were purified with the NucleoSpin® Clean-Up kit using the NTC buffer and resulting DNA libraries were freeze dried and frozen prior usage for next selection cycle. Detailed selection conditions are summarised in Supp. Table S2.

### Next Generation Sequencing

After 10 selection cycles, samples from all cycles for both tumour and kidney of the two DNA libraries were prepared for next generation sequencing (NGS) analysis on Illumina HiSeq1500 platform following the protocol from Tolle *et al*.^78^. Shortly, a first PCR with index containing primers was performed. Those indexes allow the analysis of 12 different samples on the same round. After purification of the PCR product as described above, up to 12 different samples with different indexes were mixed with equal amounts of DNA, to a final amount of 2 µg DNA. Then, addition of adapter sequences by enzymatic ligation was performed according to the manufacturer by using TruSeq DNA PCR-Free Sample Preparation Kit LT (Illumina), following the steps “End Repair”, “Adenylation” and “Adapter Ligation”. Samples were then purified via agarose gel (2%) and silica based spin-columns, and eluted in resuspension buffer. Quantitative PCR was performed for library validation with the KAPA library quantification kit (Sigma-Aldrich) prior sequencing. Seventy-five base pair single end sequencing was carried out. Raw NGS data was analysed using the COMPAS (COMmonPAtternS) software.

### *In vivo* Planar NIR fluorescence imaging of candidate aptamers

Mice were housed under standard conditions with food and water ad libitum but using chlorophyll free diet 15 days before imaging in order to reduce autofluorescence signal of the animals. Imaging experiments and analysis were performed using a fluorescence Diffuse Optical Tomography (fDOT) imaging system as previously described^79^. Basically, the acquisition of Fluorescence reflectance imaging (FRI) is based on the excitation of fluorophores by the LEDs (emitting light between 650 and 670 nm) placed above the animal and on the reception of the fluorescence signal using the CCD camera and a band-pass filter (730 ± 15 nm). The CCD camera is focused at the top surface of the animal. Prior to intravenous injection and imaging of the aptamers, solutions of all sequences containing 2 nmol were prepared in DPBS with calcium and magnesium. All sequences were then heated for 3 minutes at 80 °C, spin down, let to cool in ice for 3 minutes and store at room temperature. Prior to imaging, mice were anesthetised with 4% isoflurane gas. Afterwards the level of isoflurane concentration was lowered down to 2–2.5%. The natural auto-fluorescence of the mice was recorded just before injection and was further subtracted in order to obtain the accurate fluorescence signal from the injected fluorescent probes. Then, the fluorescent aptamers were injected in the tail vein using a 29 G (insulin-type) syringe in a volume of 100 µL. Fluorescence images of dorsal side view were acquired 5 min, 90 min and 180 min post injection. From experience, good contrast is obtained after exposition times of a few milli-seconds. Since aptamers are rapidly eliminated by the urinary pathway, the biodistribution of aptamers in prostate tumours could not be measured by *in vivo* imaging. Therefore, animals were euthanised 3 h after injection and organ resection permitted *ex vivo* fluorescence analysis of tumours and muscles.

### Planar Image analysis

For the semi-quantitative analysis of fluorescence planar images, the ImageJ software (http://rsbweb.nih.gov/ij/) was used. The first step was to subtract the intrinsic background noise of the camera from each image acquired. Second step was to normalise the images to the same exposure time. An ROI was manually drawn, to delineate the tumour, based on the white images (photographs) that are always acquired before initialising the experiments. The mean of intensity in this region was subtracted from the mean of intensity in the same area before injection, which corresponds to the auto-fluorescence of the animal at time t_0_. Using normalised images as well, a ROI is manually drawn for each time to delineate a reference healthy area close to the tumour tissue. The tumour targeting of aptamers was evaluated by dividing the mean fluorescence from the tumour by the mean fluorescence from the healthy zone. For *ex vivo* analysis, the same protocol was used and the tumour/muscle ratios were calculated.

### qPCR of tissue

Orthotopic and xenograft tumours from mouse injected with Alexa Fluor 680 labeled D3P-21 and control sequences D3P-library, D3P-4 and D3P-16 from the *in vivo* screening were homogenised and purified as described above for D3P library. The amount of extracted DNA was quantified with NanoDrop 2000C (Thermo Scientific) and for qPCR quantification samples were normalised to a same OD. Two µL sample of the extracted DNA were added to 18 µL of a PCR master mix, containing 1X GoTaq colorless buffer, 2 mM MgCl_2_, 0.2 mM dNTPs, 300 nM of non-modified reverse and forward primers, 1X SYBR Green I (Sigma Aldrich) and 2.5 U GoTaq polymerase. Thermal conditions were optimised to 10 min 95 °C followed by 40 cycles of 30 s at 95 °C, 30 s at 64 °C and 45 s at 72 °C. Thermal cycling was performed in an iCycler Thermal Cycler upgraded with the iQ5 real-time PCR detection system (Bio-Rad, Germany). DNA standards were included; 20–0.002 fmol in 1 to 10 dilution. Each sample and standard were run in duplicates.

### Cell culture

For the *in vitro* evaluation of D3P-21 aptamer, different cell lines were used. The tumour cell line PC-3 was obtained from ProQinase, Ramos (Burkitt’s lymphoma), A549 (human non-small cell lung cancer) and H460 (large cell lung cancer) were obtained from ATCC (American Type Culture Collection). MCF7 cells (breast cancer) were purchased from CLS (Cell lines service). Splenocytes and PMBC’s were obtained from the spleen and the blood, respectively, of C57/BL6J mouse strain (kindly provided by Dr. Sven Burgdorf from the LIMES Institute in Bonn). Ramos, MCF7, H460 and LNCaP cells were cultured in RPMI 1640 medium while PC-3 and A549 cells were cultured in DMEM, high glucose, GlutaMAX^TM^ supplemented (ThermoFisher) both with 10% fetal bovine serum (Sigma) at 37 °C in humidified air containing 5% CO_2_, and maintained by routine passage every 2-3 days. Prior usage, cells were counted with a hemacytometer. Suspension cells were centrifuged 5 min at 200 rcf and the pellet was suspended in fresh medium to obtain a cell suspension with the desired densities. For adherent cells, 100000 cells/well in appropriate medium were seeded in 24 well plates 24 hours prior the assay and proper amount of LNCaP cells were seeded in T12.5 cell culture flasks 48 hours prior the assay.

### Binding assays

Flow cytometry assays were performed in a BD FACSCanto cytometer and qPCR assays with an iCycler Thermal Cycler upgraded with the iQ5 real-time PCR detection system (Bio-Rad, Germany).

### Flow cytometry

For studying the interaction of D3P-21 aptamer to different cancer cell lines, 3′-Alexa Fluor 680 labeled D3P-21 and control library D3P-library were used whereas 3′-Atto647N labeled oligonucleotides were used to study the 11-kDa PEG moiety influence in the aptamer binding and the interaction of D3P-21 aptamer to both prostate cancer cell lines PC-3 and LNCaP. Cells were incubated with 100 or 200 nM of D3P-21 aptamer or D3P-library control in 200 µL of binding buffer or 750 µL for LNCaP cells (DPBS with 0.49 mM MgCl_2_, 0.9 mM CaCl_2_ and 0.5 mg/mL salmon sperm) for 30 minutes at 37 °C and 5% CO_2_. Then, cells were washed 3 times with washing buffer (DPBS with 0.49 mM MgCl_2_, 0.9 mM CaCl_2_) *via* centrifugation at 200 g for 5 minutes at room temperature for suspension cells, and with scraping of the adherent cells in the last washing step followed by centrifugation for volume reduction. For LNCaP cells, CaCl_2_ was removed from the last washing step in order to prevent clumping of the cells. For each measurement, 10000 cells were analysed in the flow cytometer. The data was analysed using FlowJo software.

### qPCR

The same incubation protocol as used for flow cytometry analysis for the individual sequences/library was followed. After 3 washing steps, cold ddH_2_0 was added and cells were incubated at 4 °C for 30 min. Cells were then recovered from the well plate, heated at 95 °C for 5 min, and diluted to 5 cells/µL for analysis *via* qPCR. qPCR protocol was identical to the one described above (qPCR of tissue section).

### Stability of D3P-21 sequence

Two µM D3P-21 aptamer either bearing or lacking the 11-kDa PEG moiety in the 5′-end were incubated in human serum and DPBS containing calcium and magnesium at 37 °C. Samples were collected at different time points (0, 1, 3 and 18 hours) and intact DNA was quantified with qPCR as described above. Human serum was kindly provided by Dr. Jens Müller from the University Hospital Bonn.

### CD Spectroscopy studies

CD spectra were recorded at 20 °C with a Jasco J-810 spectrophotometer. The measurements were performed with 8 µM of DNA oligos in water, PBS without potassium (130 mM NaCl, 7 mM Na_2_HPO_4_·H_2_O and 3 mM NaH_2_PO_4_·2H_2_O, pH 7.4), and increasing concentrations of KCl (0.1, 1, 4 and 10 mM). The spectra were recorded with 100 nm min-1 scanning speed and 4 accumulations.

### TNF-α HTRF assay

The TNF-α homogeneous time-resolved fluorescence (HTRF) assay was performed in accordance with the manufacturer guidelines (Cisbio). Briefly, immortalised murine embryonic stem cell-derived macrophages in 96-well plates were treated with increasing concentrations of D3P-library and aptamers D3P-21 and D3P-20, both containing or lacking of the 5′–11 kDa PEG moiety, for 24 hours. CpG oligonucleotide and LPS were used as positive controls. The cell supernatants were collected and stained with anti-TNF-α antibodies conjugated to FRET molecules. Changes in the fluorescence emission spectrum were proportional to the TNF-α concentration.

## Supplementary information

Supplementary Information
