# Supplementary material for: Targeting hormone refractory prostate cancer by in vivo selected DNA libraries in an orthotopic xenograft mouse model
Source: Sci Rep. 2019 Mar 21;9:4976. doi: 10.1038/s41598-019-41460-2 (PMC6428855; doi:10.1038/s41598-019-41460-2)
Supplement: Supplementary file 1 — Supplementary Information [file 41598_2019_41460_MOESM1_ESM.pdf]

Supplementary information

Targeting hormone refractory prostate cancer by *in vivo* selected DNA libraries in an orthotopic xenograft mouse model

Laia Civit[1], Ioanna Theodorou[2], Franziska Frey[1], Holger Weber[3,10], Andreas Lingnau[3,11], Carsten Gröber[4], Michael Blank[4], Chloé Dambrune[2], James Stunden[5], Marc Beyer[6,7,8], Joachim Schultze[6,7], Eicke Latz[5], Frédéric Ducongé[2], Michael H.G. Kubbutat[3,10], and Günter Mayer[1,9]*

[1] Chemical Biology and Chemical Genetics, Life and Medical Sciences (LIMES) Institute, University of Bonn, and Centre of Aptamer Research and Development, University of Bonn, Gerhard-Domagk-Str. 1, 53121 Bonn, Germany

[2] CEA, DSV, I2BM, Molecular Imaging Research Center (MIRCen), 18 Route du Panorama, 92260 Fontenay-aux-Roses, France

[3] KTB Tumorforschungsgesellschaft mbH, Research Division ProQinase, Breisacher Str. 117, 79106 Freiburg, Germany

[4] AptaIT GmbH, Am Klopferspitz 19a, 82152 Planegg-Martinsried, Germany

[5] Institute of Innate Immunity, University Hospital Bonn, Sigmund-Freud-Str. 25; 53127 Bonn, Germany

[6] Genomics and Immunoregulation, Life and Medical Sciences (LIMES) Institute, University of Bonn, Carl-Troll-Straße 31, 53115 Bonn, Germany

[7] Platform for Single Cell Genomics and Epigenomics at the DZNE and the University of Bonn, Sigmund-Freud-Str. 27; 53127 Bonn, Germany

[8] Molecular Immunology in Neurodegeneration, German Center for Neurodegenerative Diseases (DZNE), Sigmund-Freud-Str. 27, 53127 Bonn, Germany

[9] Center of Aptamer Research and Development (CARD), University of Bonn, Gerhard-Domagk Str. 1, 53121 Bonn, Germany

[10] current address: ProQinase GmbH, Breisacher Straße 117, 79106 Freiburg, Germany

[11] current address: Genmab B.V., Yalelaan 60, 3584 CM Utrecht, The Netherlands

Supplementary Figure 1

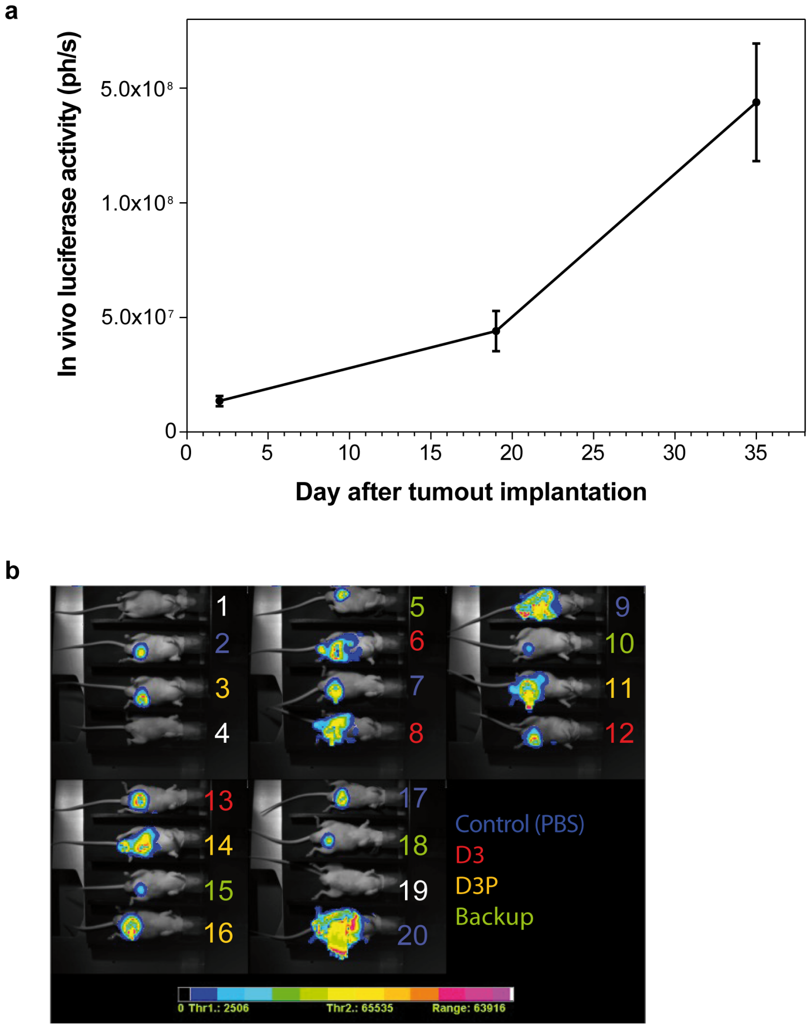

Figure S1: Bioluminescence imaging for the monitoring of the orthotopic tumour growth. (**a**) On Day 0, 3x10^6 PC-3-Luc tumour cells in 15µl PBS were implanted orthotopically into 20 male NMRI nude mice. During the course of the study, tumour growth was monitored *in vivo* on days 2, 19 and 35 using bioluminescence imaging. For this purpose, 150 mg/kg D-Luciferin was injected intraperitoneally (*i.p.*) into the mice 7 min before anesthetisation. Light emission was measured 10 min post injection with a CCD-camera for 5 min using a NightOWL LB 981 bioluminescence imaging system (Berthold Technologies, Germany). The *in vivo* luciferase activity (ph/s) with SEM is shown. (**b**) Example of a batch of mice used for one selection cycle of both D3 and D3P. Tumours of mice that were injected with PBS were used as control during the DNA extraction and amplification.

Supplementary Figure 2

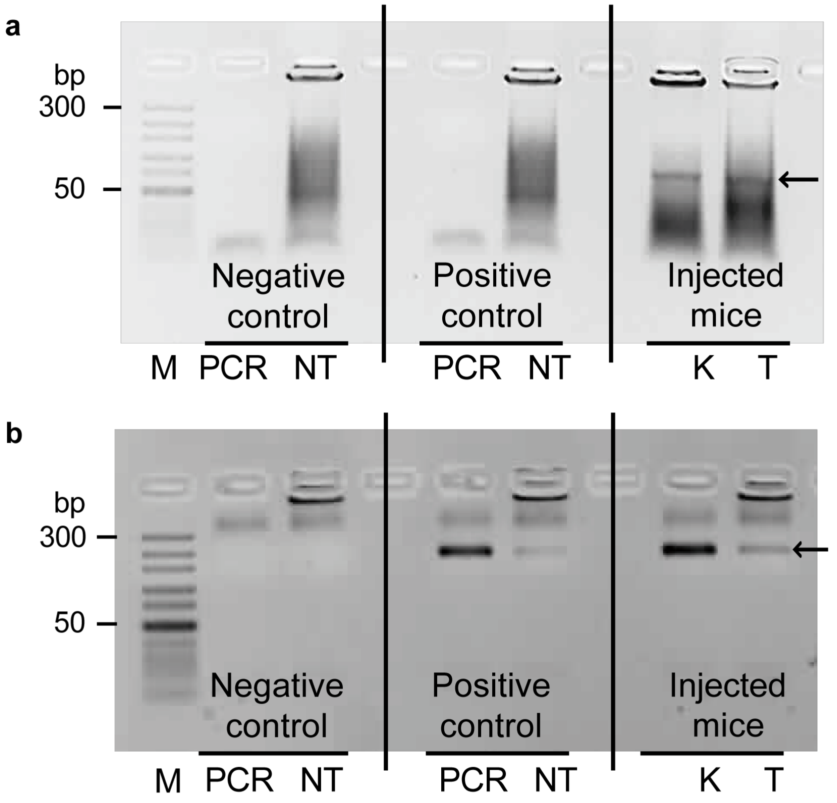

Figure S2: Agarose gel analysis of the PCR amplified DNA from the kidneys (K) and tumours (T) recovered from library injected mice. Tumours from mice injected with PBS (NT (negative tumour)) were also subjected to the same homogenisation and purification process and used as a negative control (for the control of the specificity of the amplified DNA extracted from the tumours (T)). Spiked NT with 0.2 fmol of the initial libraries were used in order to control possible inhibition of the amplification (positive control NT). Positive and negative PCR controls were also included (PCR). (a) Example of a selection cycle (selection cycle 2) from the D3 library (14 PCR cycles) and (b) D3P library (20 PCR cycles). Extracted RNA is visualised in the D3 corresponding gel as smear. An ultralow range DNA ladder was used (M). Arrows indicates the expected dsDNA D3 and D3P bands. The amount of required PCR cycles was empirically determined by PCR cycling, analysing and dependending on the results adding further PCR cycles to enhance product or stopping the reaction.

Supplementary Figure 3

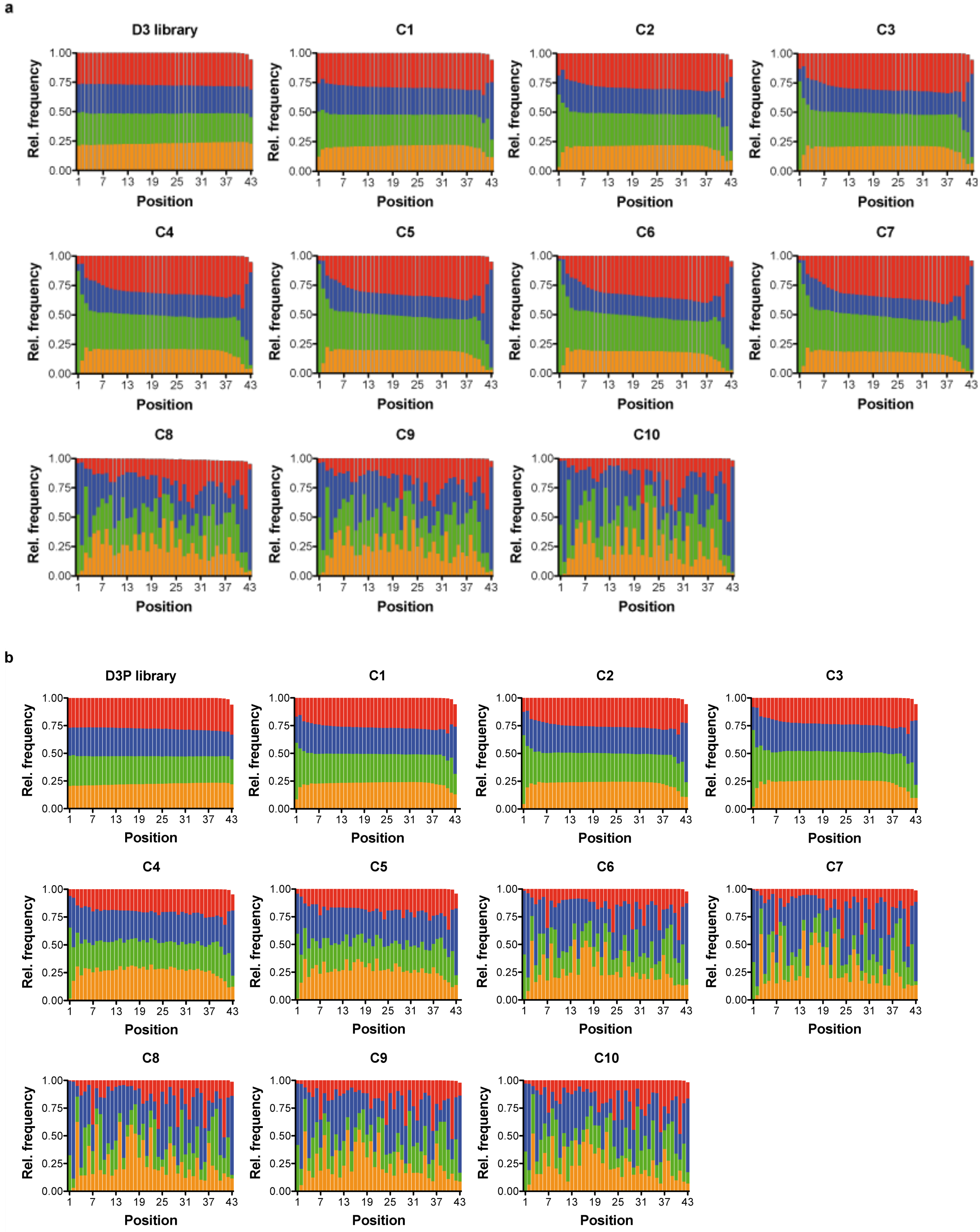

Figure S3: Next generation sequencing analysis. The distribution of nucleotides over the 43 nucleobases of the initial random region of the starting library and the DNA libraries form the selection cycles C1 to C10 of the *in vivo* selection using D3 (**b**) and the distribution of nucleotides over the 43 nucleobases of the initial random region of the starting library and the

DNA libraries from selection cycles C1 to C10 of the *in vivo* selection using D3P (**c**). Orange: dA, green: dC, blue: dG, and red: dT.

Supplementary Figure 4

a

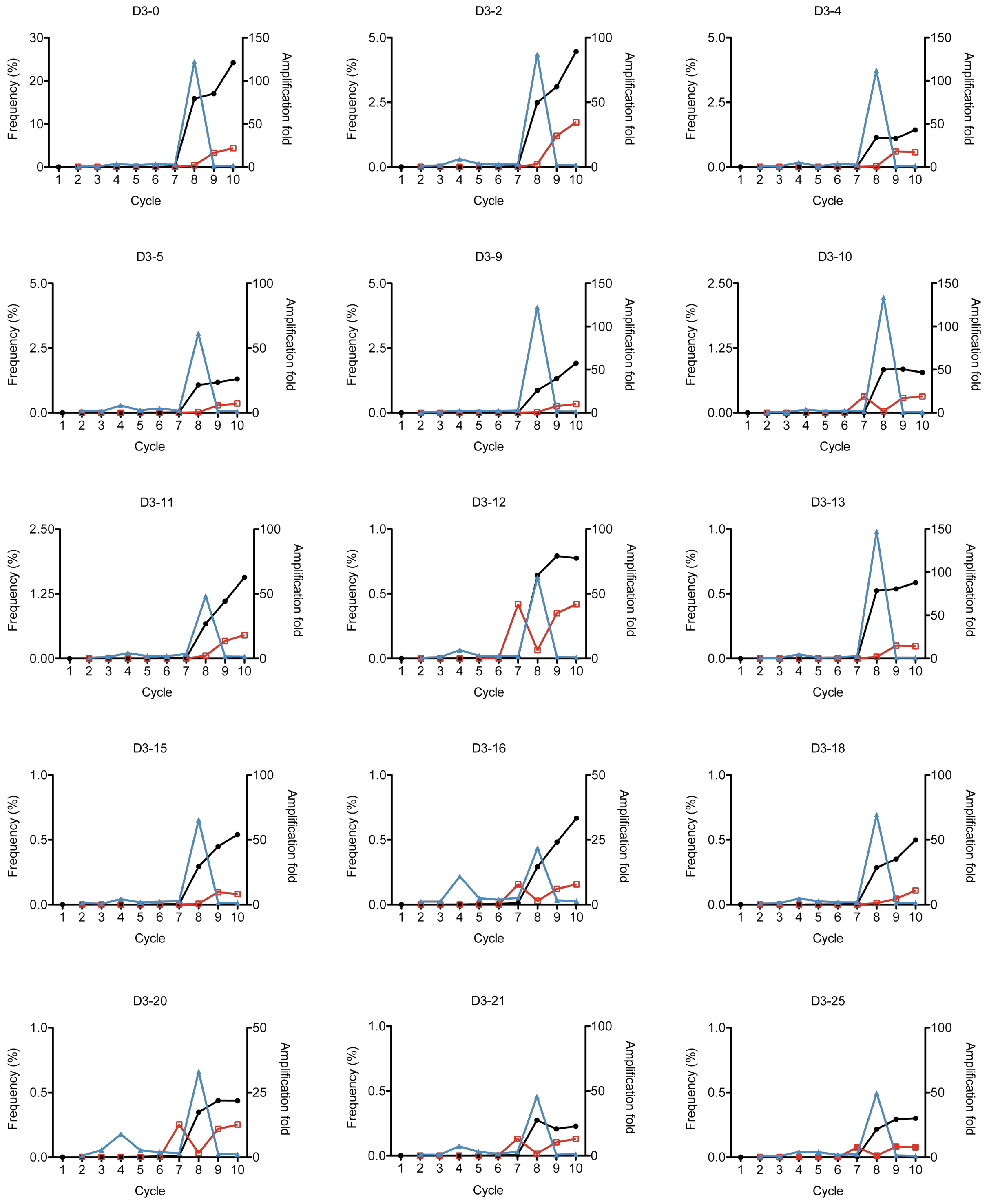

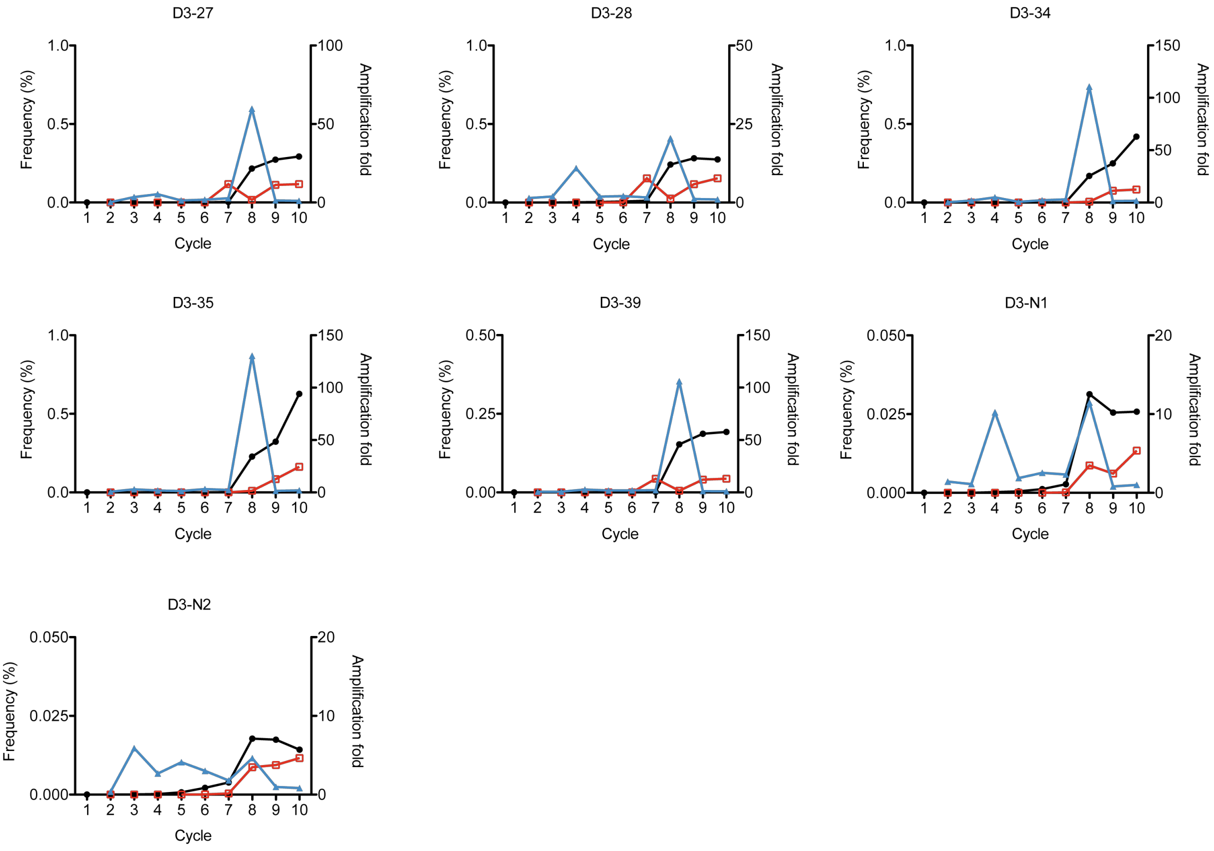
D3-27
D3-28
D3-34
D3-35
D3-39
D3-N1
D3-N2
Frequency (%)
Amplification fold
Cycle
1 2 3 4 5 6 7 8 9 10
1.0
0.5
0.0
0.50
0.25
0.00
0.050
0.025
0.000
150
100
50
25
20
10
0

b

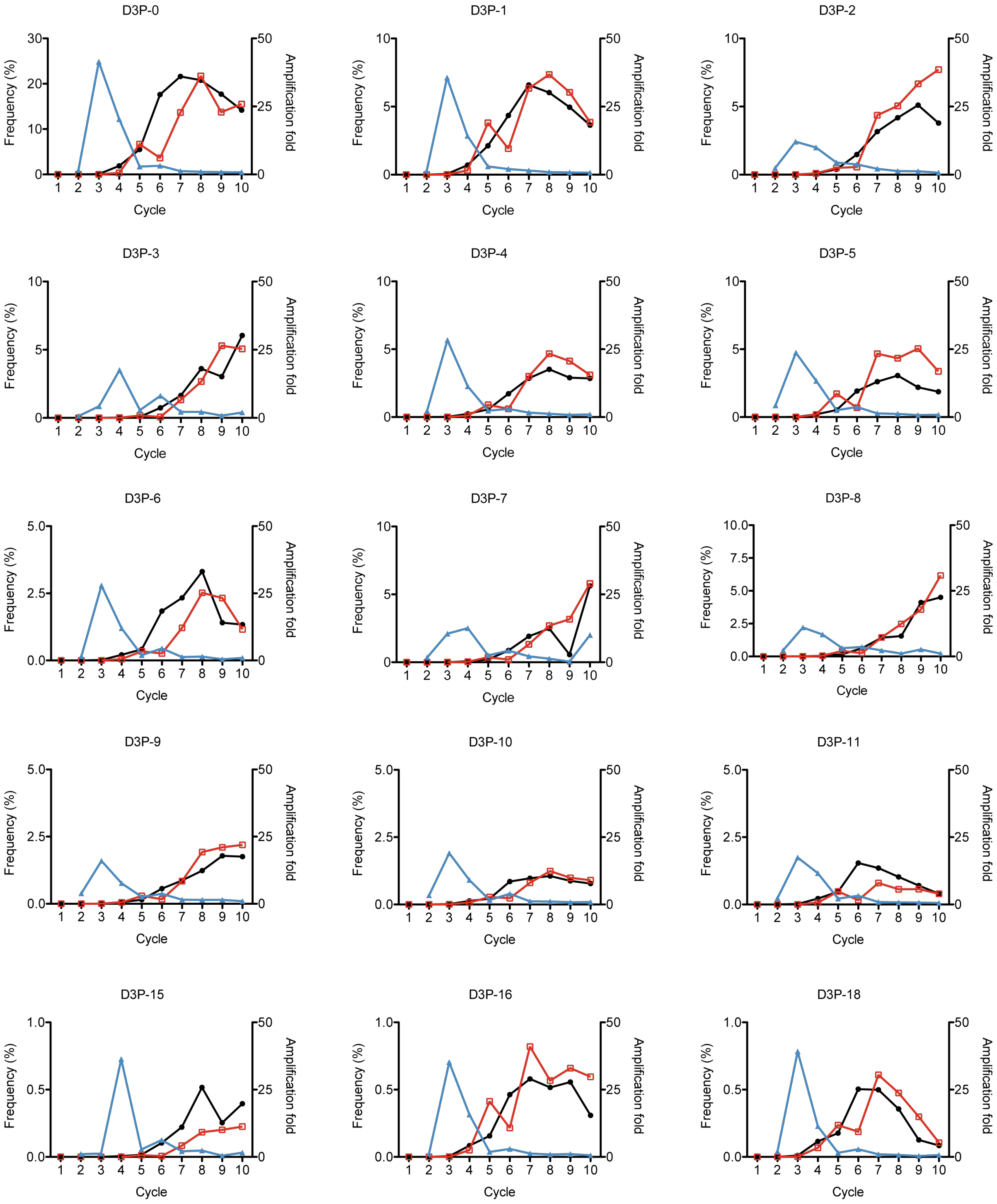
D3P-0
D3P-1
D3P-2
D3P-3
D3P-4
D3P-5
D3P-6
D3P-7
D3P-8
D3P-9
D3P-10
D3P-11
D3P-15
D3P-16
D3P-18
Frequency (%)
Amplification fold
Cycle

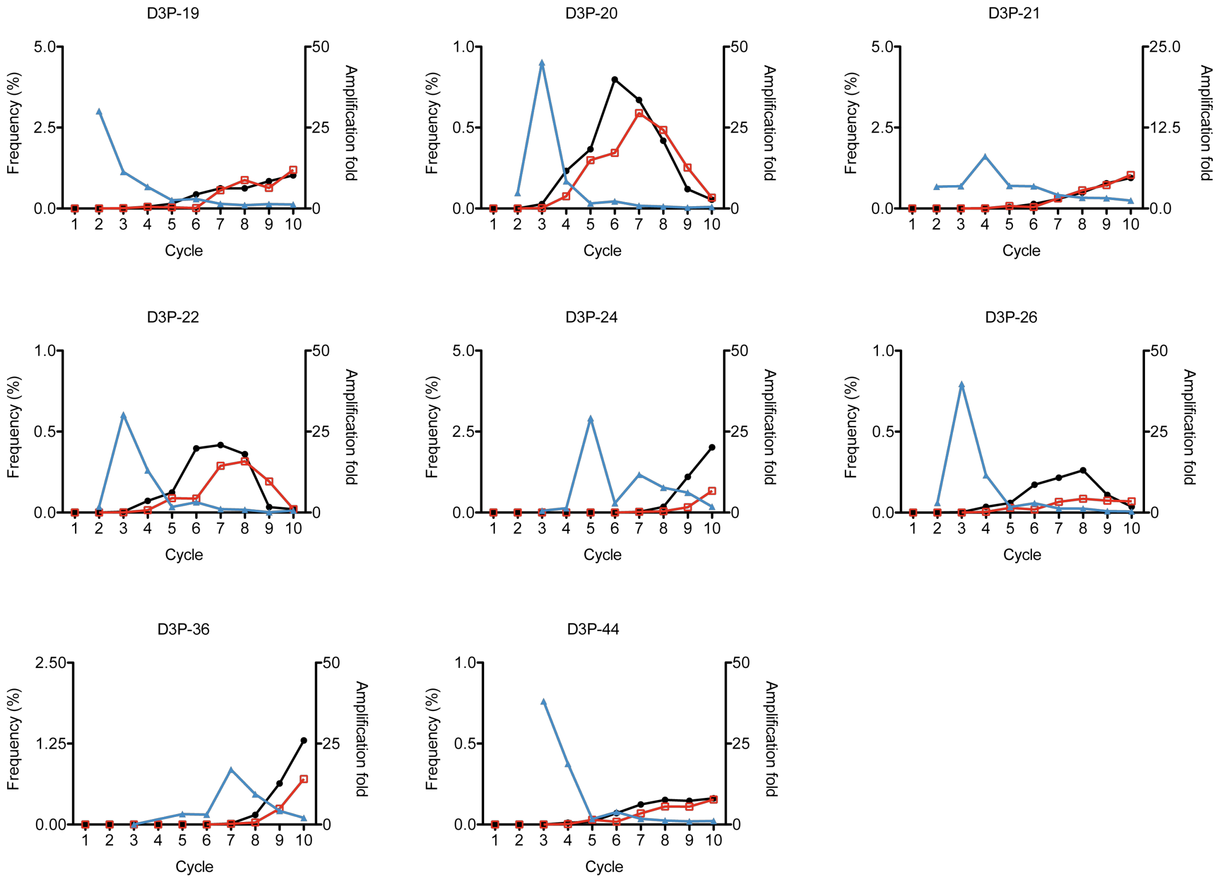

Figure S4: Next-generation sequence analysis of the individual oligodeoxynucleotides obtained from the *in vivo* selection experiements. The frequency of the selected sequences among the selection cycles recovered from the tumour tissue (black) or the kidneys (red) and the corresponding amplification-fold profile of the sequences recovered from the tumour tissue (blue) for (**a**) D3-related and (**b**) D3P-related sequences.

Supplementary Figure 5

a

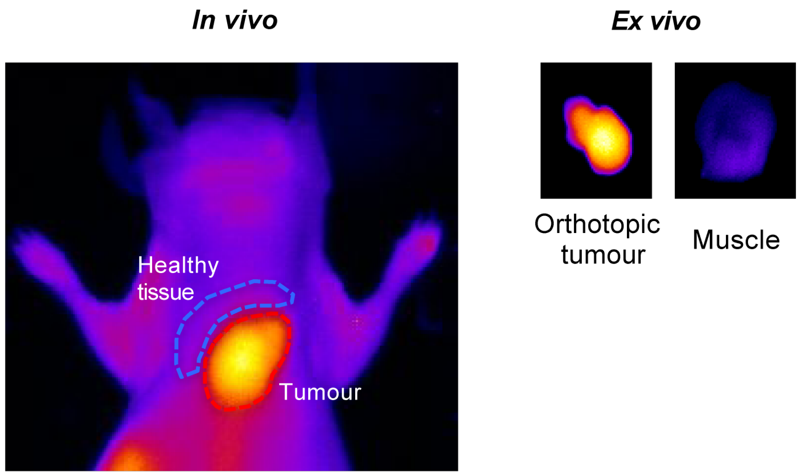

b

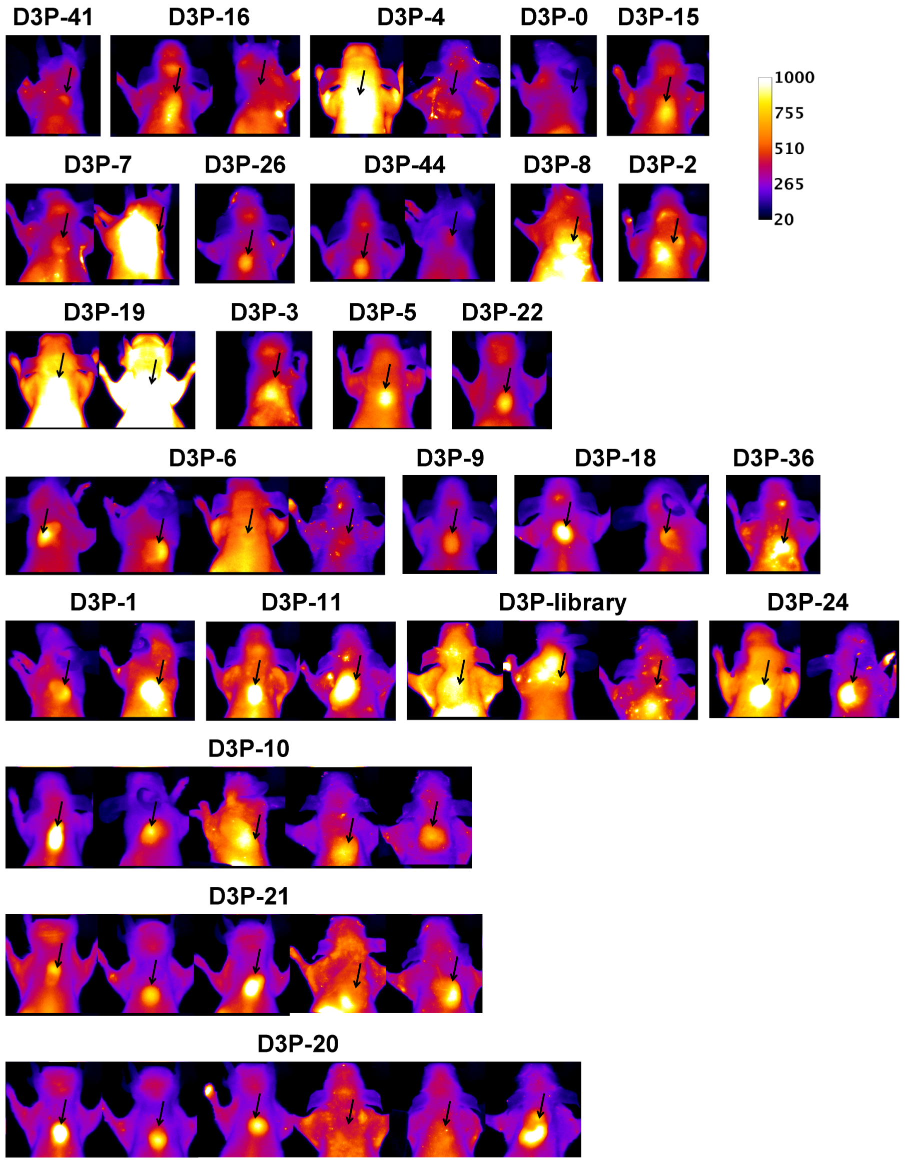

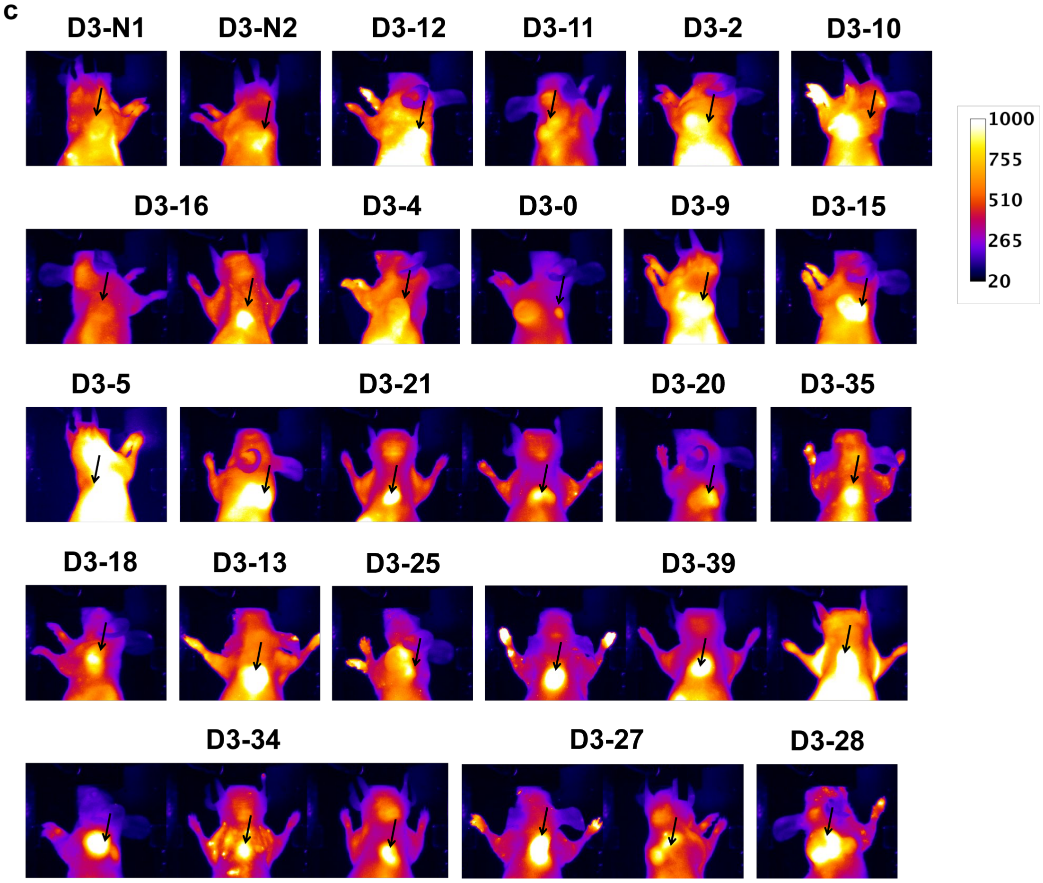

Figure S5: Planar imaging of analysed sequences. (**a**) Example of a fluorescence planar image of a xenograft PC-3-LN tumour 180 min post injection with the indicated Alexa Fluor 680 labeled oligonucleotide. The illustrations show the regions of interest (ROI) for tumour (in dark red) and normal tissue (dark blue) used for the calculation of the mean fluorescence intensity. (**b**) Dorsal view of the *in vivo* imaging of mice injected with 2 nmol of the selected sequences evolved from *in vivo* selection experiments using D3P or (**c**) D3 180 min post injection (exposure time 1000 ms).

Supplementary figure 6

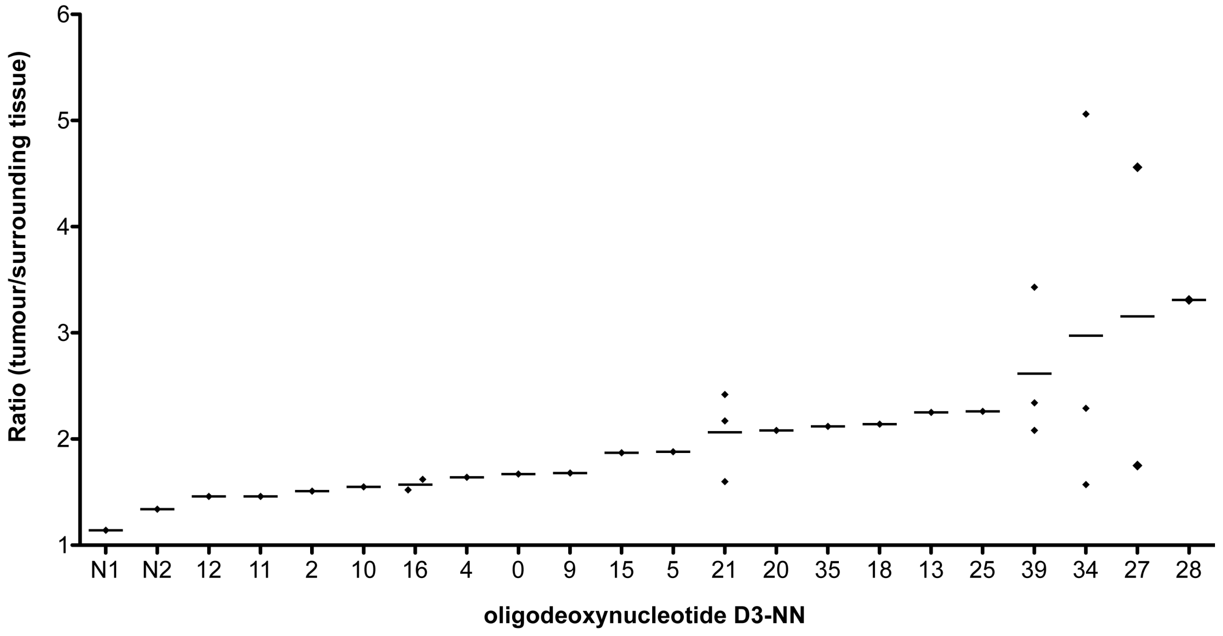

Figure S6: Planar imaging of D3 *in vivo* SELEX sequences. Ratio of the fluorescence signals of tumour and surrounding tissue of the indicated sequences.

Supplementary figure S7

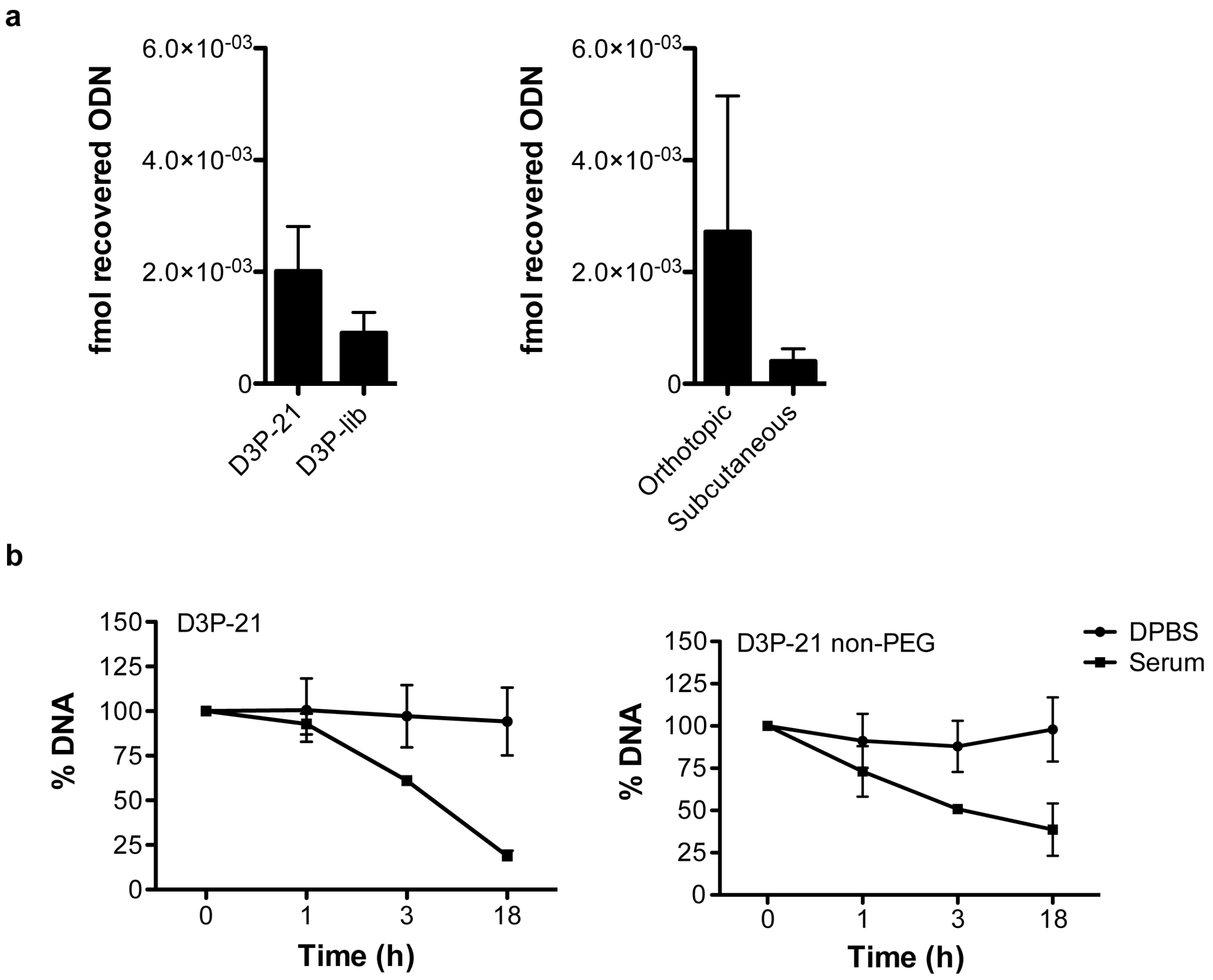

Figure S7: Evaluation of D3P-21 properties *in vitro*. (**a**) Quantitation of D3P-21 or control D3P-library targeting subcutaneous or orthotopic PC-3 tumours extracted from mice used in the *in vivo* screening. The relative amount of the aptamer and native library of tumours from only subcutaneous mouse models (right) and aptamer D3P-21 from both orthotopic and subcutaneous tumours (left) was quantified *via* qPCR. Data is normalised to the OD of the sample and is represented as the mean value between tumours injected with the same oligonucleotide (n = 4, 2 independent experiments). (**b**) Stability of D3P-21 aptamer in human serum compared to DPBS with Ca^{2+}/Mg^{2+}. 2 μM of pegylated and non-pegylated D3P-21 was incubated at 37ºC in human serum (■) or buffer (●) for 0, 1, 3 and 18 h. Quantification of the intact aptamer was assessed by qPCR and data is represented as % of intact DNA.

Supplementary Figure S8

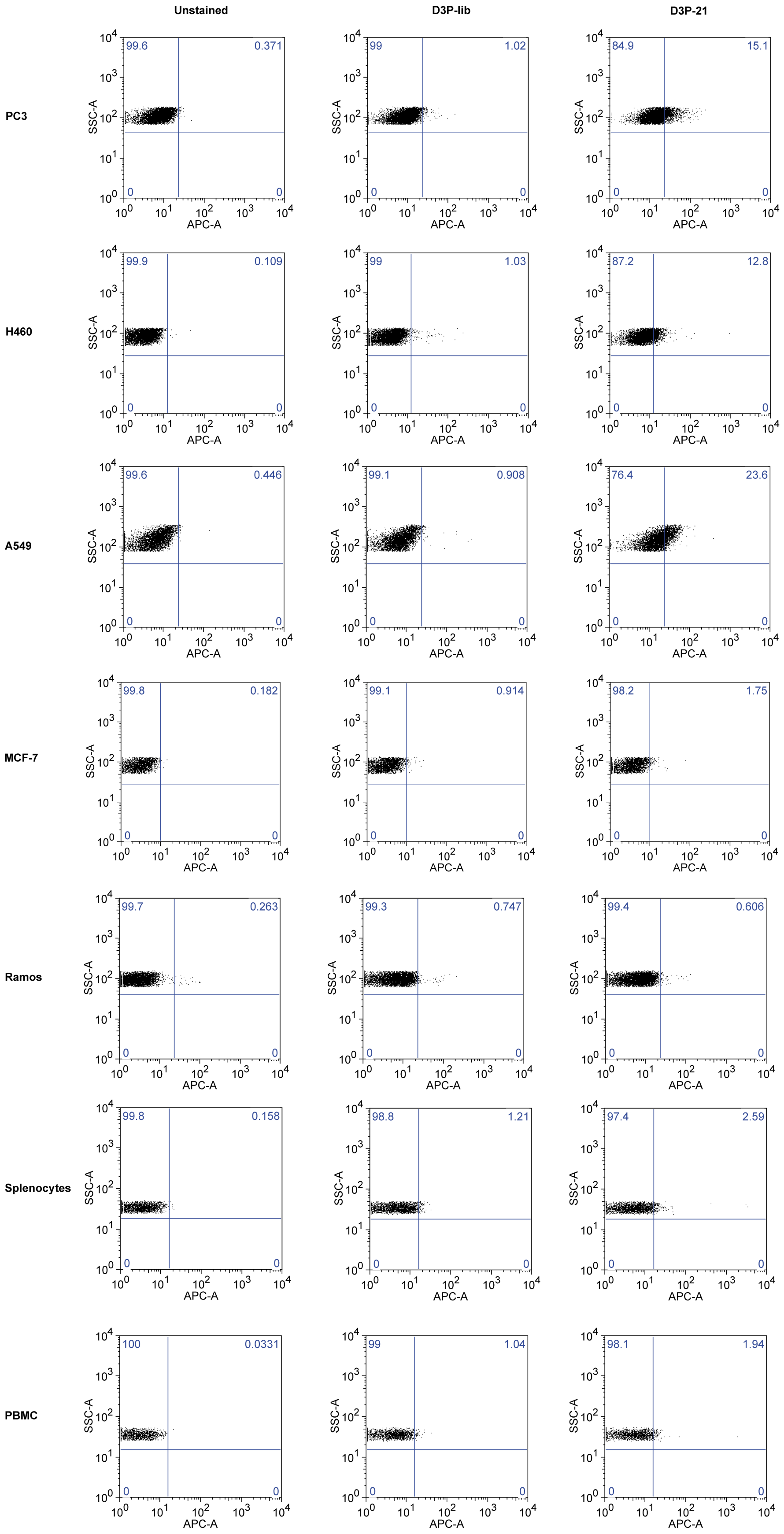

Figure S8: Evaluation of D3P-21 properties *in vitro*. Representative scatter plots from the flow cytometry analysis of the interaction of aptamer D3P-21 and the DNA library D3P (D3P-lib) with prostate cancer PC-3 cells, murine splenocytes, and murine peripheral blood mononuclear cells (PBMC) and other cancer cell lines (MCF7, H460, A549, and Ramos).

Supplementary Figure S9

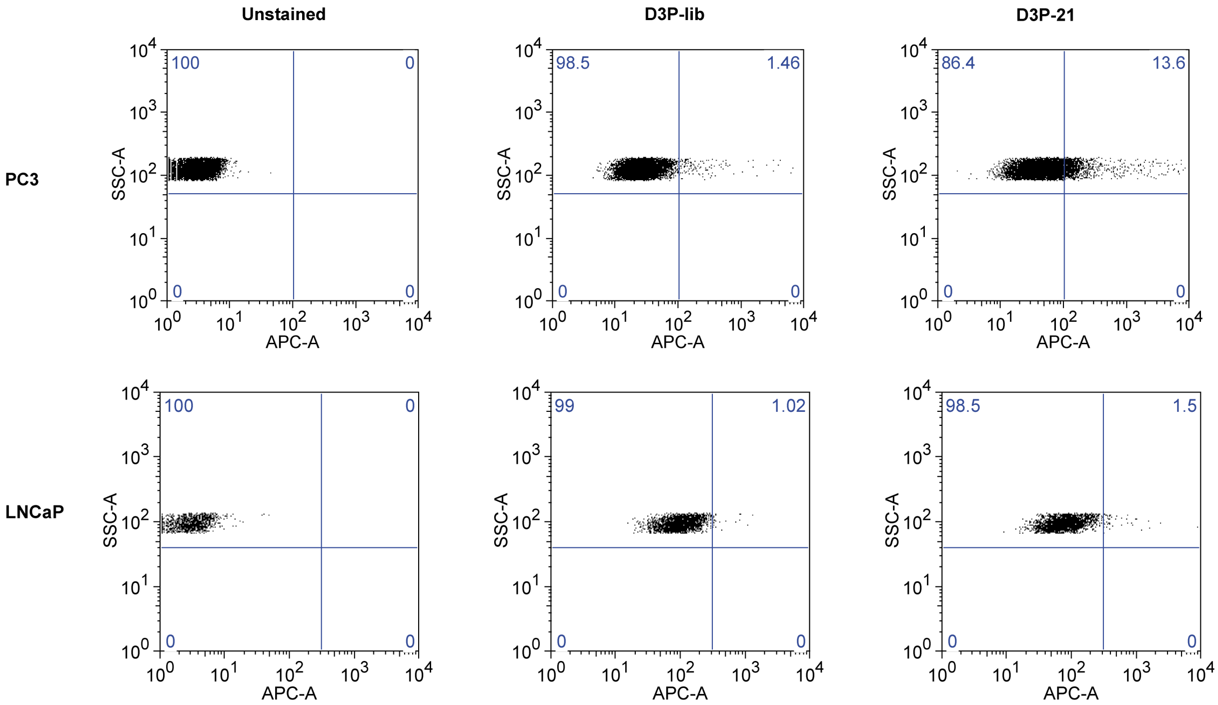

Figure S9: Evaluation of D3P-21 properties *in vitro*. Representative scatter plots from the flow cytometry analysis of the interaction of aptamer D3P-21 and the D3P-lib with prostate cancer PC-3 and LNCaP cells.

Supplementary Figure S10

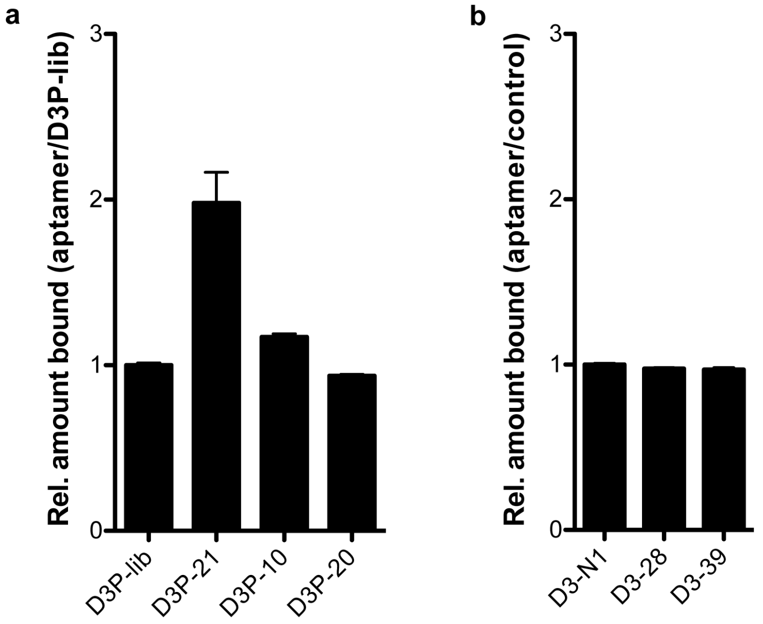

Figure S10: *In vitro* evaluation of other D3P and D3 sequences. *In vitro* flow cytometry assay to monitor the aptamer candidates D3P-21, -10 and -20 and D3-28 and D3-39 and the naïve D3P-library binding to prostate cancer PC-3 cells. Represented is the ratio of binding of (**a**) D3P-21, -10 and -20 in respect to the D3P-library and (**b**) D3-28 and D3-39 in respect to D3-N1 as control. Cells were incubated with 200 nM of the aptamer labelled in the 3'-end with Alexa Fluor 680. Represented as mean ± SD (n = 4, 2 independent experiments).

Supplementary Figure S11

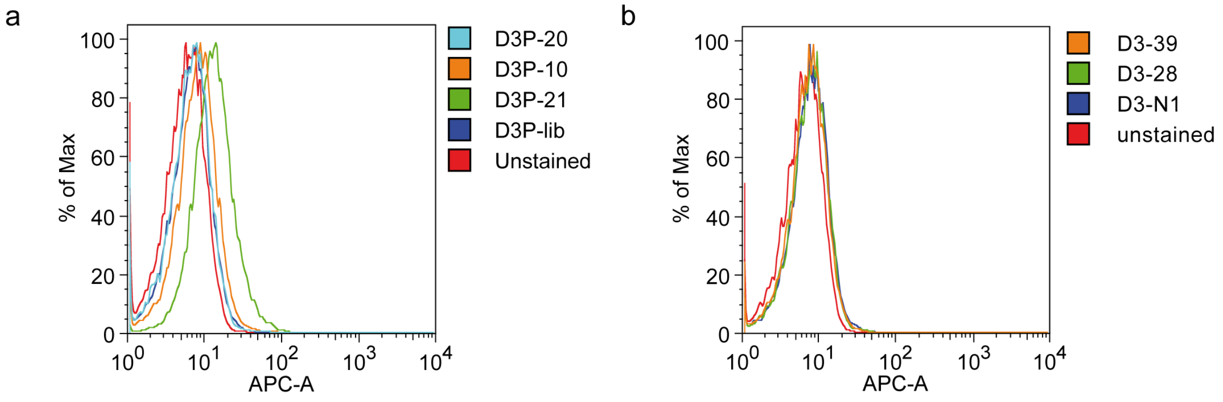

Figure S11: *In vitro* evaluation of other D3P and D3 sequences. Representative histograms overlays of the binding to prostate cancer PC-3 cells (**a**) for aptamers D3P-21, -10, -20 and the D3P-library and (**b**) D3-28, D3-39 and D3-N1 as control.

Supplementary Figure S12

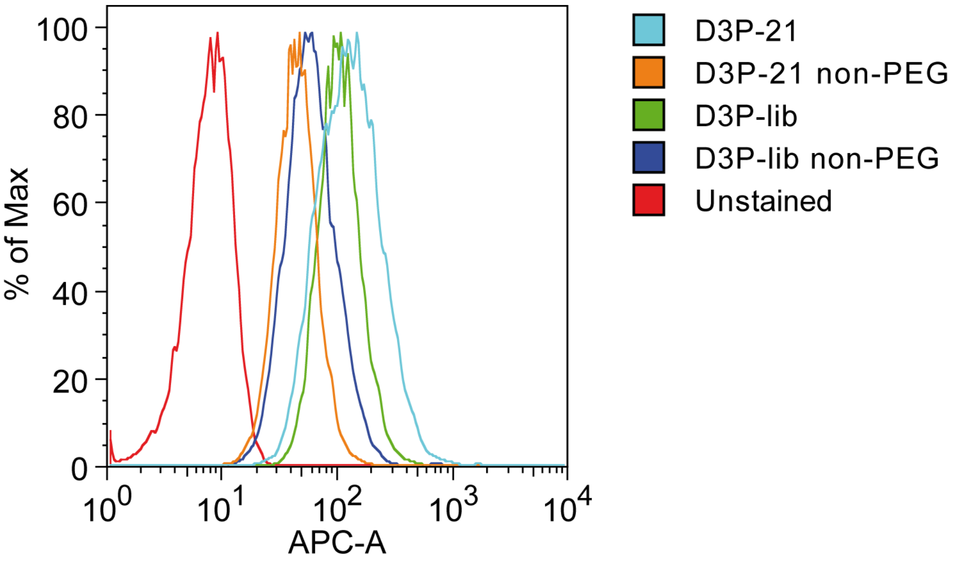

Figure S12: Evaluation of D3P-21 properties *in vitro*. Representative histogram from the flow cytometry analysis of the impact of the PEG moiety of D3P-21 on its interaction properties.

Supplementary Figure S13

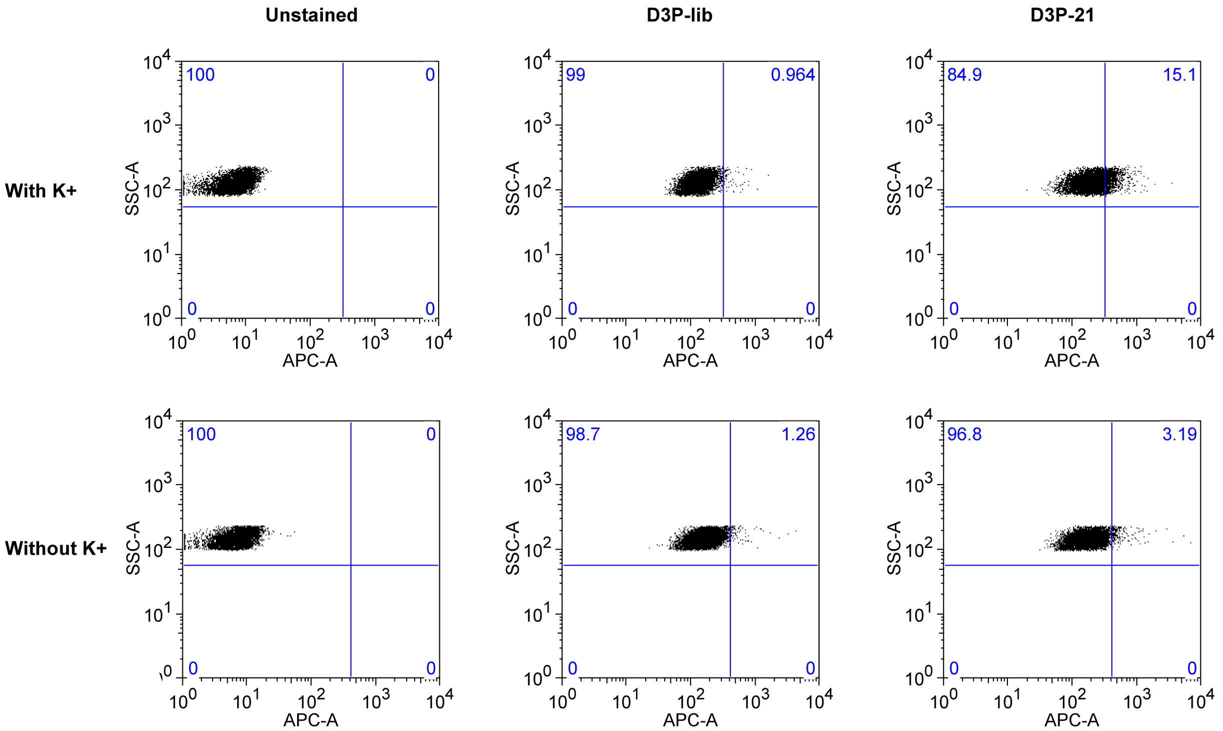

Figure S13: Impact of potassium ions on the interaction D3P-21 with PC-3 cells. Representative scatter plots from the flow cytometry analysis to monitor the influence of potassium in the binding of aptamer D3P-21 to PC-3 cells.

Supplementary Table S1: Weight of the tumours and kidneys extracted during the *in vivo* selection. For each selection cycle, an average of 3 tumours and kidneys from 3 mice were used. Exceptions are for the control tumour and selection cycle 1, where 1 to 3 tumours were used depending disponibility.

Selection cycle	D3 SELEX (mg)			D3P SELEX (mg)		
	Tumour	Kidney	Control Tumour	Tumour	Kidney	Control Tumour
1	190<br>140	490	70.0	100<br>270<br>70	924.0	170.0
2	298.5<br>192.3<br>245.8	225.7<br>350.4<br>364.8	106.6<br>200.9	73.4<br>118.1<br>28.4	296.1<br>245.7<br>371.0	70.3<br>35.0<br>30.1
3	78.0<br>320.4<br>287.8	245.6<br>336.4<br>240.8	195.0	159.4<br>286.3<br>388.6	285.0<br>286.3<br>308.4	254.8
4	185.0<br>309.0<br>156.0	277.0<br>182.9<br>297.0	233.5	210.0<br>147.0<br>421.0	236.4<br>315.3<br>262.5	276<br>135
5	352.0<br>118.0<br>252.0	310.0<br>281.0<br>304.0	286.0<br>107.0	262.0<br>163.0<br>285.0	230.0<br>262.0<br>262.0	184.0<br>105.0
6	90.0<br>289.0<br>139.3	328.0<br>280.0<br>305.0	229.0	102.0<br>34.0<br>35.0	251.0<br>287.0<br>273.0	173.0
7	147.0<br>244.7<br>183.0	243.3<br>298.0<br>283.7	518.0	163.1<br>60.0<br>255.0	263.0<br>290.9<br>207.0	286.5
8	71.3<br>78.7<br>26.3	266.8<br>226.0<br>327.0	137.3	77.2<br>265.4<br>141.8	227.4<br>295.7<br>244.9	148.3
9	134.2<br>174.5<br>123.1	291.3<br>268.2<br>267.9	96.8	163.8<br>106.1<br>96.8	232.3<br>229.8<br>255.8	148.2
10	185.6<br>78.9<br>76.5	269.7<br>250.9<br>259.4	49.5<br>45.3	128.8<br>172.8<br>52.0	248.6<br>277.0<br>257.4	141.4

Supplementary Table S2: Given is the amount of DNA library injected into the tail vein of each mouse, the amount of tumour tissue and kidneys recovered, and the number of PCR cycled needed to amplify the corresponding DNA libraries after recovery and work up. In selection cycle 8, the PCR amplification cycle of D3 library extracted from the tumour and the kidney could not be determined.

Sel. cycle	Library inj. (nmol)	PCR amplification cycle			
		D3 Tumour (15 mg/PCR)	D3 Kidney (15 mg/PCR)	D3PEG Tumour (6.25 mg/PCR)	D3PEG Kidney (6.25 mg/PCR)
1	5	22	22	22	22
1	0.1	16	20	20	18
2	2	14	14	18	16
3	1	12	12	18	14
4	0.5	10	10	19	18
5	0.1	12	12	16	14
6	0.1	10	10	18	16
7	0.1	12*	10*	14	14
8	0.1	ND†	ND†	14	14
9	0.1	12*	12*	14	12
10	0.1	12*	12*	16	14

** 6.25 mg/PCR † not determined*

Supplementary Table S3: Total number and the number of unique sequences identified by NGS of the DNA libraries of the *in vivo* selection experiments using D3 and D3P that were extracted from the tumour tissue and the kidney after each selection cycle.

Selection	D3 Tumour		D3 Kidney		D3P Tumour		D3P Kidney	
cycle	Total	Unique	Total	Unique	Total	Unique	Total	Unique
Library	836905	833437	836905	833437	692693	688961	692693	688961
1	6550671	6481171	ND*	ND*	3961402	3503741	8187382	3010465
2	9137535	8899766	6473798	6283787	6577458	5003632	3852313	3034743
3	12425013	11540566	847505	836728	11406435	6579862	4340316	3241497
4	4054136	3740905	847505	836728	4231809	2196563	4465426	1850191
5	8028226	6253539	167392	164703	6525846	2505151	4322350	1411286
6	6053048	4308913	2922399	2298589	7757549	1171435	4944222	2267647
7	4580172	3200837	4552304	3116828	2820205	320025	2593169	464686
8	6177817	970662	4640161	1754336	5239351	298547	4116600	234815
9	4305255	666647	7459475	1514455	1833526	128842	3958717	188224
10	2335749	259580	4682352	1144214	409262	36940	1932907	92215

**ND: not determined*

Supplementary Table S4: Random region of the selected sequences identified by NGS analysis of D3 *in vivo* SELEX and their frequency at selection cycle 10.

Name	Sequence random region	Frequency tumour (%)	Frequency kidney (%)
D3-0	GGCGAACACGGCGGAGACACTACAGTCTTCGACCGGCACGGTG	24.23	4.39
D3-2	CGCAGACGTGTCCACGGTGAGACTGATAATCGGGTGGCGAACG	4.47	1.73
D3-4	GGACGTTCACAGGGAGACACCTGTTACATTGCTCCGATTAGGT	1.43	0.57
D3-5	GGGGACGCTCAGCGGAGGCAAAATGCAGTGGTCCAGGGCCTGG	1.31	0.36
D3-9	CGCATGGAAGGCGCATTAGCGGGACAAGACCAGGAAACCCCGT	1.92	0.34
D3-10	CGCGTAAGCGGTCAATCATCACGCAGCGAGACGAGCATACGGG	0.78	0.31
D3-11	GGAGGCGAGACTGCGAGGCAAGCGCTTACAGGGAAACAACGTG	1.57	0.45
D3-12	GGGGTTACTCCAGCGAGACAACCAGGCGTGGTCAAGAAGTTGG	0.78	0.42
D3-13	GGACGAAACAGCAGCGGAGACATACGTCTAATCGCAAGGCGAG	0.59	0.10
D3-15	GGACGAACTACAGCGTCTTATCTGGAGCAAATACAACGCCTCG	0.54	0.08
D3-16	GGCGGCCGCAGTGAGAGACTGATATATTGTCGGACACCGTTCG	0.67	0.16
D3-18	CCGTAGGGACGCGAGGCTGTCCACAGCGGAGACTATTTCGGCC	0.50	0.11
D3-20	GGGGGCACTACGCTCAGGAGACTCAATACTCGGCACCGGTGGG	0.44	0.25
D3-21	GGAGGCAACGGAGCGGAGACATTGACTGAGTGAACGTGTAGTG	0.23	0.13
D3-25	GGTAGTGAAACTAGCGAGACAACTAGCTGGTCTACATCGTGGG	0.30	0.08
D3-27	GGATGGCACCGACGGGGACAAGAAGTAAGTCTCCGAGTATCGG	0.29	0.12
D3-28	GGAGGACCGGCGAGACATTGTGGAGTTGCGTAAACGTGTTTGG	0.27	0.15
D3-34	GGACGAACCAGGGAGACGTATAGCTAGTCTTGCGCAGCAAACG	0.42	0.08
D3-35	CGCAGTGGAACACGGCGAGACAAACGGCATCGGTCGCGTAGTG	0.63	0.16
D3-39	GGACGAAACCCCAGCGAGACAAAGAATACATCTAGATATATCG	0.19	0.04
D3-N1	GGACAACACGGCGACCCCATCGAGACCCGGAGACATGTCGGTG	0.026	0.013
D3-N2	CACCGGAGAGCGATACCCTGTCCTGAGGGATTCCATCCATGTG	0.014	0.011

Supplementary Table S5: Random region of the selected sequences found by NGS analysis of D3P *in vivo* SELEX and their frequency at selection cycle 10.

**Name**	**Sequence random region**	**Frequency tumour (%)**	**Frequency kidney (%)**
D3P-0	GGAGCATACGGGGAGAAAGAGACTGGAGTCGTGGGTACCGTCG	14.20	15.50
D3P-1	CGCGGGTGTGAGCAGGACATGATTACGAGGCAGAGGGAACTGA	3.64	3.86
D3P-2	CGAATCGAAGATGAGGCCGAGGAGAGGAGACCAAGGATAGAGG	3.79	7.71
D3P-3	GGAGGGACATAGCGGAGACTATACGTCAGCTCTCGTTTCGAGT	6.05	5.07
D3P-4	GGATCGGACAGCGAGGGAAGTACGGTGGGATGCCGCTCGGTAT	2.85	3.10
D3P-5	GGAGGGCAGCAAAGGAGACCTCGAGAGATATGGATGGCCCCAG	1.87	3.37
D3P-6	GGATCGCACTGCCAAGATCGACTGGCATAAGCCCGCGGCTGTG	1.34	1.16
D3P-7	GGCGAACACGGCGGAGACACTACAGTCTTCGACCGGCACGGTG	5.63	5.80
D3P-8	GGCGGCATGTACAGCGACAAGACGGTGGACATGAGTAATCGTG	4.50	6.17
D3P-9	GGTGGCCACGGCAGACAGATATGCGTGCTCCGTCGCGAGATGG	1.76	2.20
D3P-10	GGAGACGCATAGCAGACAAGACTTTAACGGGGGCCATTCTGTG	0.78	0.90
D3P-11	CCGAACGTTGCAAAGAACATCGAGACAGACAAGCAACCCTCGG	0.40	0.40
D3P-15	GGAGCACATAGGAGCAAGCATACTTAGCATGCTACGAAGTAGG	0.40	0.23
D3P-16	CGACACGCAGTAGAGTTGAACGGATCCTGGAGCAAGGAACGGG	0.31	0.60
D3P-18	CGCAAGGGCATATCAGCAGACAGATCAAGATGTCGTGTCTCAC	0.08	0.11
D3P-19	CGCAGAGGGGCGAAACAGCCAAGATCGGGGTCCATTGTGATGG	1.03	1.20
D3P-20	GGATCATACCGGCCGAGATGCGATGCGACGTGACGTAGTTCAG	0.05	0.07
D3P-21	GGAAAGAGCACGGCCAAGTCAGGGGGAATCGACTACGTCGGGG	0.94	1.04
D3P-22	GGAGCGAACGGCAGACAGGTTACGAGCGTTCCTTGAGAATCAA	0.02	0.02
D3P-24	CACCGGCCTAACATATTCCTATCTACCTTACCACACCTTGTCC	2.01	0.67
D3P-26	GGGTCAGACAGGAGACACGAAGACGCGACAGGCGCCAGGCTGG	0.04	0.07
D3P-36	CCACGCGCCCAGTCCAGCCCCCCCCATGGGTCTTATATGTACC	1.30	0.70
D3P-44	CGCGGGACAAACAGCGGAGACTTTGATGACACTGAGGCCCTCG	0.16	0.15
